# Gut metagenome profile of the Nunavik Inuit youth is distinct from industrial and non-industrial counterparts

**DOI:** 10.1038/s42003-022-04372-y

**Published:** 2022-12-24

**Authors:** Jehane Y. Abed, Thibaud Godon, Fadwa Mehdaoui, Pier-Luc Plante, Maurice Boissinot, Michel G. Bergeron, Richard E. Bélanger, Gina Muckle, Natalia Poliakova, Pierre Ayotte, Jacques Corbeil, Elsa Rousseau

**Affiliations:** 1 Centre de Recherche en Infectiologie de l’Université Laval, Axe Maladies Infectieuses et Immunitaires, Centre de Recherche du CHU de Québec-Université Laval, Québec City, QC Canada; 2 Centre de Recherche en Données Massives de l’Université Laval, Québec City, QC Canada; 3 Département de microbiologie-infectiologie et d’immunologie, Faculté de médecine, Université Laval, Québec City, QC Canada; 4 Département d’informatique et génie logiciel, Université Laval, Québec City, QC Canada; 5 Centre Nutrition, Santé et Société (NUTRISS), Institute of Nutrition and Functional Foods (INAF), Université Laval, Québec City, QC Canada; 6 Axe santé des populations et pratiques optimales en santé, Centre de recherche du CHU de Québec-Université Laval, Hôpital du Saint-Sacrement, Québec City, QC Canada; 7 Département de pédiatrie, Faculté de médecine, Université Laval, Québec City, QC Canada; 8 Centre mère-enfant Soleil, CHU de Québec-Université Laval, Département de pédiatrie, Québec City, QC Canada; 9 École de psychologie, Faculté des sciences sociales, Université Laval, Québec City, QC Canada; 10 Centre de Toxicologie du Québec, Institut national de santé publique du Québec (INSPQ), Québec City, QC Canada; 11 Département de médecine sociale et préventive, Faculté de médecine, Université Laval, Québec City, QC Canada; 12 Département de Médecine Moléculaire, Faculté de médecine, Université Laval, Québec City, QC Canada

**Keywords:** Microbiome, Metagenomics

## Abstract

Comparative metagenomics studies have highlighted differences in microbiome community structure among human populations over diverse lifestyles and environments. With their unique environmental and historical backgrounds, Nunavik Inuit have a distinctive gut microbiome with undocumented health-related implications. Using shotgun metagenomics, we explored the taxonomic and functional structure of the gut microbiome from 275 Nunavik Inuit ranging from 16 to 30-year-old. Whole-metagenome analyses revealed that Nunavik Inuit youths have a more diverse microbiome than their non-industrialized and industrialized counterparts. A comparison of k-mer content illustrated the uniqueness of the Nunavik gut microbiome. Short-chain fatty acids producing species, and carbohydrates degradation pathways dominated Inuit metagenomes. We identified a taxonomic and functional signature unique to the Nunavik gut microbiome contrasting with other populations using a random forest classifier. Here, we show that the Nunavik Inuit gut microbiome exhibits high diversity and a distinct community structure.

## Abstract (web summary)

Nunavik Inuit gut microbiome retains a high intra-individual and lower inter-individual diversity, and is distinct from the gut microbiome of non-industrial and industrial populations

## Introduction

The gut microbiome has co-evolved with its human host^1,2^. Thus far, research studies consistently show that this co-evolution is mainly driven by the host lifestyle and environment^3–6^. A large body of literature suggests that in recent human history, the industrialization of lifestyle was a critical factor in shaping the human gut microbiome^1,2,6^. Lifestyle industrialization (also referred to as westernization or urbanization) is a complex process that occurred progressively over the last 100–200 years^2^, characterized by modified dietary habits, sedentarization, and increased access to medication (e.g., antibiotics) and sanitation^7^. Using cross-sectional comparative studies, researchers have profiled the human gut microbiome of rural non-industrialized and urban industrialized populations spanning various geographical locations^8–14^. These studies identified specific signatures of microbiome industrialization, including loss of intra-individual diversity (alpha diversity), loss of particular taxa, and increased inter-individual diversity (beta diversity). The distinctive microbial signature of the industrialized population results possibly from a combination of lifestyle factors, such as dietary habits.

Thus far, most investigated non-industrial populations rely on a foraged fiber-rich diet with little animal products and have limited access to modern medicine. By contrast, industrialized populations’ diets consist mainly of food from a globalized food chain, low in fiber and high in fat^5–7^. Consequently, little data is available on the gut microbiome profiling of individuals with diets rich in naturally sourced animal products and was limited to small groups of Nunavut Inuit (15 and 16 Nunavut Inuit), whose gut microbiomes were profiled using 16 S rRNA amplicon sequencing^15,16^.

Approximately 12,000 Inuit live in the 14 communities of Nunavik, a vast territory located north of the 55^th^ parallel in the province of Quebec. Their genome architecture seems unique because they have little admixture with other present-day populations, and differences among villages correlate with their migration routes^17^. Inuit inhabit circumpolar regions, and the Arctic environment has drastically influenced their way of life and genetics^17^. Traditional Inuit land-based activities include plant/berry picking, hunting terrestrial and marine mammals, fishing, and harvesting seaweed and seafood^18^. These activities closely relate to the land because of food seasonality. Country food items (also referred to as traditional food) are mostly consumed raw, dried or fermented (e.g., beluga whale parts) and are nutrient-dense and excellent sources of protein, omega-3 polyunsaturated fatty acids, selenium, vitamins and other nutrients^18,19^. Due to different historical events, government policies, and increasing contact with other indigenous and non-indigenous groups, the last seventy years were characterized by sedentarization and increased market food consumption^20^. Inuit dietary patterns are also impacted by climate change and food insecurity^18^. However, country foods still represent an important part of the Inuit diet and seem to gain popularity among Inuit youths^21^.

We hypothesized that the Inuit gut microbiome composition would be unique based on the abovementioned characteristics and the Inuit way of life. Therefore, we profiled the gut microbiome of 275 young Inuit and additionally profiled the gut microbiome of 4 other subjects living in the same region. All participants were between the age of 16 and 30 years old. We used shotgun metagenomic sequencing to characterize the microbiome and compare it with populations following non-industrial (*n* = 73)^7,9,13^ and industrial (*n* = 104) lifestyles in different environments^10,22–24^. We found that the Nunavik Inuit gut microbiome has retained a high intra-individual diversity and lower inter-individual diversity, similar to characteristics of non-industrial populations^8^. Furthermore, we show that the genomic microbiome content in Nunavik Inuit is distinct from other previously studied populations.

## Results

### Nunavik microbiome profiling

The Data Management Committee of the *Qanuilirpitaa?* (In English: How are we now?, abbreviated Q2017) 2017 Nunavik Inuit Health Survey granted us access to the data and biological samples necessary to conduct our work. Additional information on our collaboration with Inuit organizations is provided in the Methods section. 279 fecal samples (275 Inuit + 4 other ethnicities living in Nunavik) were successfully sequenced, and comparison samples were selected from the curatedMetagenomicData R package^25^. Shotgun sequencing data obtained from Nunavik and comparison stool samples were profiled using MetaPhlAn 3.0 and HUMAnN 3.0^26^ (Supplementary Figure 1). In the combined quality-controlled datasets (Nunavik and comparison groups), 1,125 taxa (15 phyla, 29 classes, 47 orders, 92 families, 228 genera and 714 species) (Supplementary Table 1) and 26,046 pathways were detected.

### The Nunavik gut microbiome is diverse and unique

**Fig. 1:**
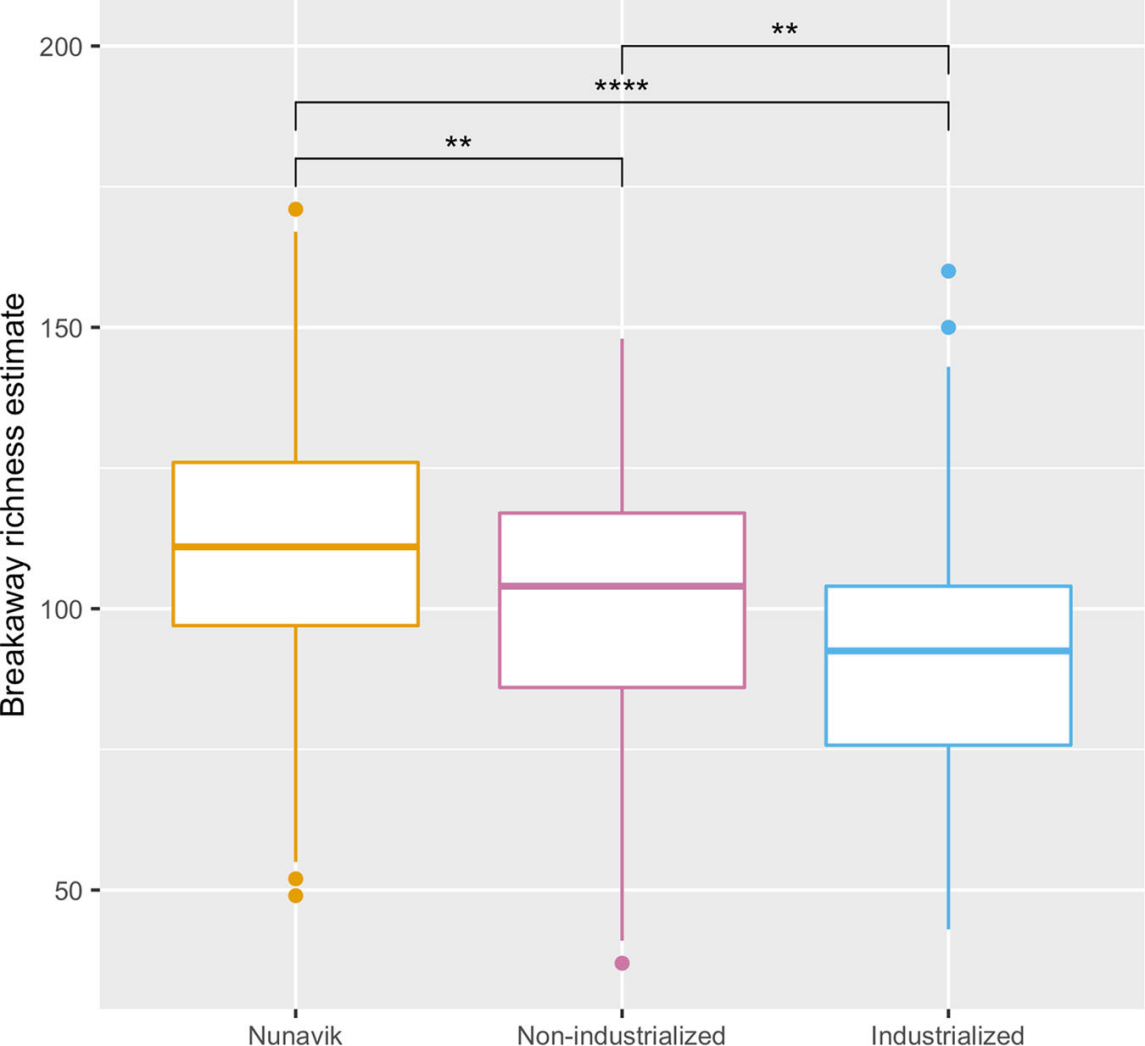
Measure of intra-individual diversity at species level of the microbiome in Nunavik Inuit, in individuals from non-industrial and industrial societies. Breakaway richness estimate highlights a higher alpha diversity in the Nunavik gut microbiome (*n* = 279), compared to non-industrial (*n* = 73, T-test; *p*-value = 0.005) and industrial (*n* = 104, T-test; *p*-value = 7.6e-14) populations. (*p-value ≤ 0.05, **p-value ≤ 0.01, ***p-value ≤ 0.001, ****p-value ≤ 0.0001).

Intra-individual diversity in the Nunavik gut microbiome is significantly higher than in non-industrial and industrial comparison groups using species-level relative abundance. This was observed with the breakaway richness estimate developed for high diversity environments^27^ (T-test; non-industrialized; *p*-value = 0.005, industrialized; *p*-value = 7.6e^−14^) as well as Shannon–Wiener (Wilcoxon-Mann-Whitney; non-industrialized; *p*-value = 1.4e^−13^, industrialized; *p*-value = 7.8e^−6^) and Simpson indices (Wilcoxon-Mann-Whitney; non-industrialized; *p*-value = 1.4e^−15^, industrialized; *p*-value = 1.6e^−6^) (Fig. 1, Supplementary Fig. 2).

We found significant differences in unknown reads percentage from MetaPhlAn 3.0 taxonomic assignation in Nunavik gut microbiome and comparison groups (Supplementary Fig. 3). In Nunavik samples, MetaPhlAn 3.0 estimated that a median of 56.3% of species are unknown, compared to non-industrialized (median unknown = 85.2%) and industrialized groups (median unknown = 64.4%).

**Fig. 2:**
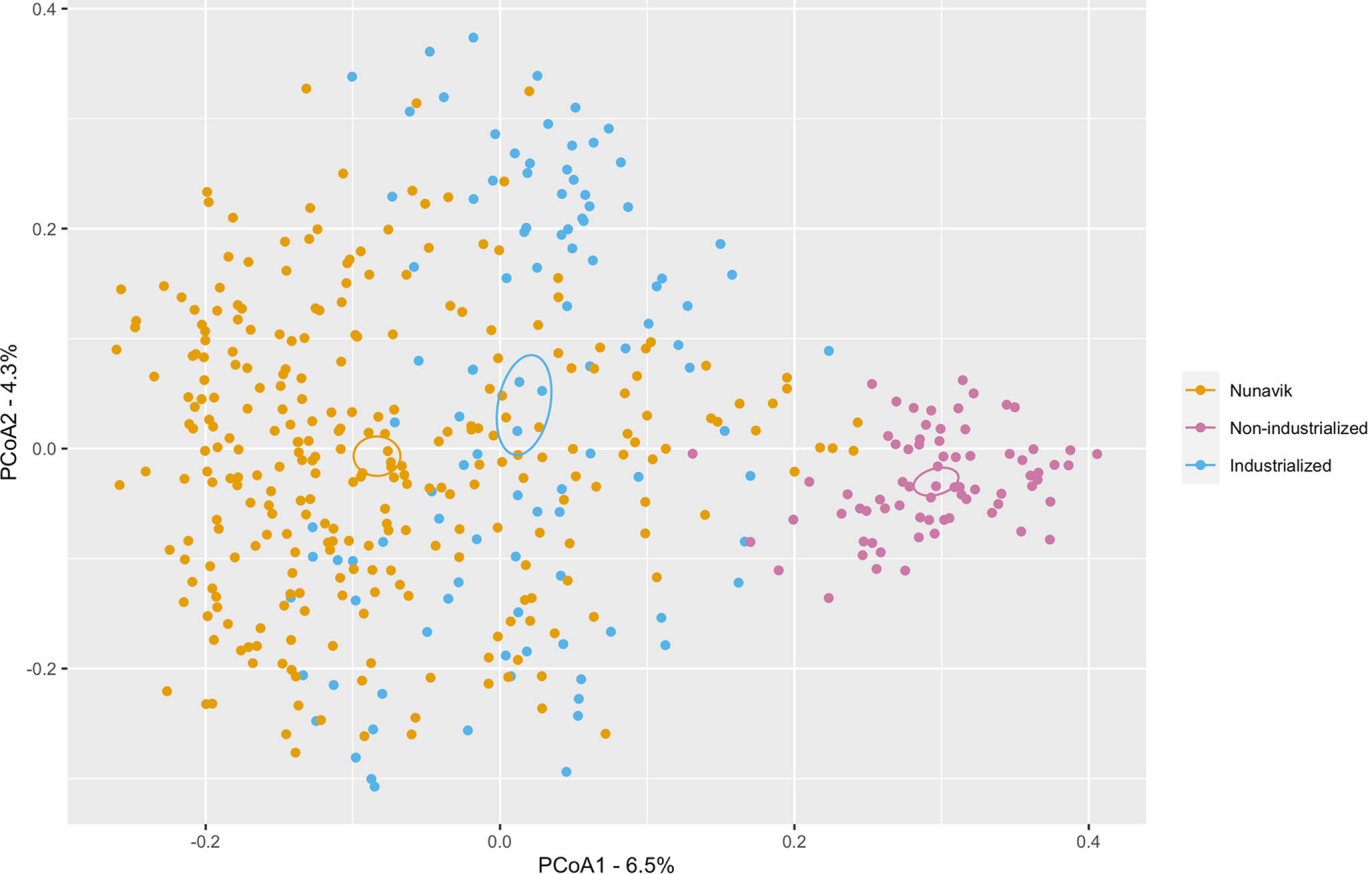
PCoA on Bray-Curtis dissimilarity of the microbiome in Nunavik Inuit, in individuals from non-industrial and industrial societies. PCoA on Bray-Curtis dissimilarity shows significant separation between species composition from Nunavik, compared to non-industrial (*n* = 73) and industrial (*n* = 104) populations. Ellipses represent the barycenter of the sample groups with their 95% confidence interval. PERMANOVA was used to evaluate significance of lifestyle (*R*^2^ = 0.06431, *p*-value = 0.001). Age and sex had a minor effect on the observed variance (age; R^2^ = 0.00440, *p*-value = 0.001, sex; R^2^ = 0.00423, *p*-value = 0.001). Analysis of Similarities (ANOSIM) demonstrated significant differences between groups based on lifestyle (lifestyle, *R* = 0.5103, *p*-value = 0.001; age, R = −0.002, *p*-value = 0.565; sex, R = 0.028, *p*-value = 0.01).

A principal coordinate analysis (PCoA) identified significant differences in community structure between Nunavik and the two comparison groups (PERMANOVA; *R*^2^ = 0.06431, *p*-value = 0.001, Fig. 2, Supplementary Figs. 4 and 5). Additionally, the inter-individual diversity of Nunavik Inuit was lower than that of the comparison groups, suggesting the Nunavik gut microbiome to be more homogeneous than the comparison groups adhering to non-industrialized or industrialized lifestyles (Supplementary Fig. 6).

Sex could be a confounding factor, the female/male ratio in Nunavik is 2:1 and 1:1 in comparison groups. However, a PCoA on Bray-Curtis dissimilarity shows minor differences between male and female participants in Nunavik (PERMANOVA; *R*^2^ = 0.00774, *p*-value = 0.001, Supplementary Fig. 7).

**Fig. 3:**
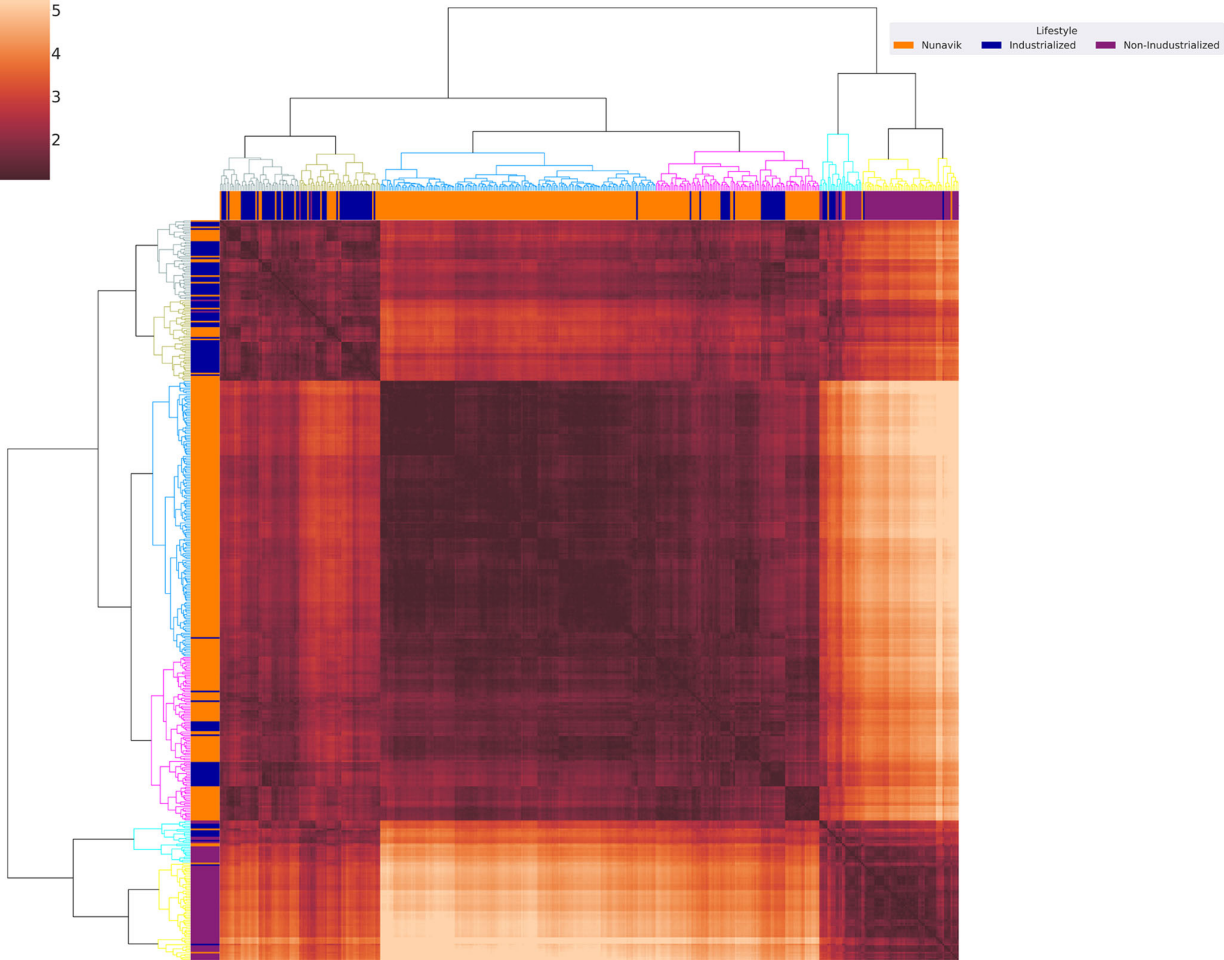
k-mer content clusters Nunavik gut microbiomes. The heatmap represents the Euclidean distance between k-mer genomic content of Nunavik and comparison samples, as computed by Ray Surveyor. Dendrograms on the X and Y axes of the heatmap represent metagenomic samples ordered based on hierarchical clustering of k-mer content distance matrix. At the leaves of the dendrograms are colored markers of each sample’s population (see legend). Within the heatmap, the darker the shade of red, the higher the similarity between metagenomes. At the top left of the heatmap, the dark red square represents the industrialized cluster and indicates that samples from the gray and khaki dendrogram branches are similar in genomic content. The two branches are composed of 69.4% and 70% of industrialized samples, respectively. Similarly, the dark red color in the center of the heatmap defines the Nunavik cluster, where the blue branch consists of 99.4%, and the pink of 76.2 % samples from Nunavik. Lastly, the bottom right square constitutes the non-industrialized cluster, with the light blue and yellow branches composed of 57.7% and 93.3% of non-industrialized samples respectively.

To determine whether identified community structure differences depended on the reference-based taxonomic profiling tool MetaPhlAn 3.0, we performed a whole metagenome comparison based on shared k-mers content^28^. Hierarchical clustering on Euclidean distance separated samples into three distinct branches representing the Nunavik, non-industrialized and industrialized clusters (Fig. 3). The Nunavik cluster comprises 90.8% of the Nunavik samples.

### Nunavik gut microbiome bacterial community

**Fig. 4:**
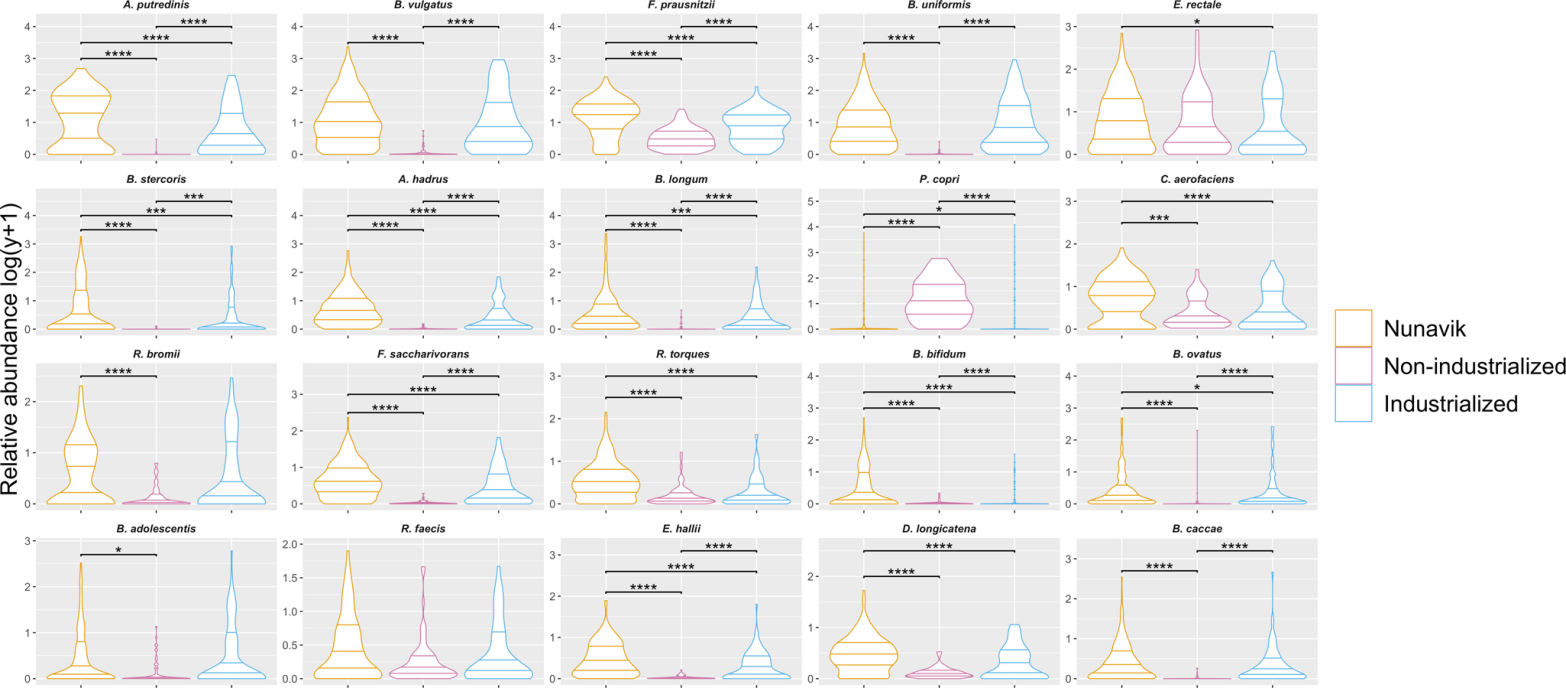
Top 20 most abundant species in the gut microbiome compared to individuals from non-industrial and industrial societies. Violin Plots represent the relative abundance (%) of the top 20 species in the gut microbiome of Nunavik youth in comparison to non-industrialized and industrialized reference populations, from most abundant to less abundant. (Wilcoxon-Mann-Whitney; *p-value ≤ 0.05, **p-value ≤ 0.01, ***p-value ≤ 0.001, ****p-value ≤ 0.0001).

Using their mean relative abundance, we identified the top 20 most abundant bacterial species in the Nunavik gut community (Fig. 4). In Nunavik, 18 out of the top 20 species were detected in at least 70% of samples (Supplementary Datas 1 and 2), and the genera *Bacteroides* and *Bifidobacterium* represented 25% and 15% of the dominant species, respectively.

Although the Nunavik diet is rich in animal products, 17 dominant species in Nunavik fecal samples were saccharolytic and produce short-chain fatty acids (SCFAs), mainly butyrate^29–39^. Notably, *Bacteroides uniformis*, *Alistipes putredinis*, *Bifidobacterium adolescentis*, *Ruminococcus bromii*, *Bacteroides stercoris*, *Bifidobacterium bifidum*, *Bacteroides caccae*, *Faecalibacterium prausnitzii*, *Ruminococcus torques*, and *Bifidobacterium longum* are all representative of the top 20 most abundant species in Nunavik metagenomes and have previously been highlighted as potential keystone species of gut microbiome resilience, which may foster recovery of homeostasis following antibiotic therapy^40^.

Consistent with previous reports, non-industrialized samples had high levels (25% of top 20 species) of *Prevotella* spp. (*P. copri, P. stercorea*) and [*Prevotella*] spp. (*Prevotella* sp AM42 24, *Prevotella* sp CAG 5226, *Prevotella* sp 885), while the industrialized gut microbiome had a high relative abundance of *Bacteroides spp*. (35% of top 20 species, *B. vulgatus*, *B. uniformis*, *B. stercoris*, *B. dorei*, *B. ovatus*, *B. caccae*, *B. eggerthii*)^41^ (Supplementary Data 1 and 2). *Prevotella* and *Bacteroides* are biomarkers of healthy microbiomes of individuals with non-industrialized and industrialized lifestyles, respectively. *Prevotella* levels increase with fiber rich, mostly plant-based diet, whereas *Bacteroides* enrichment is associated with high animal protein and fat consumption^42^.

**Fig. 5:**
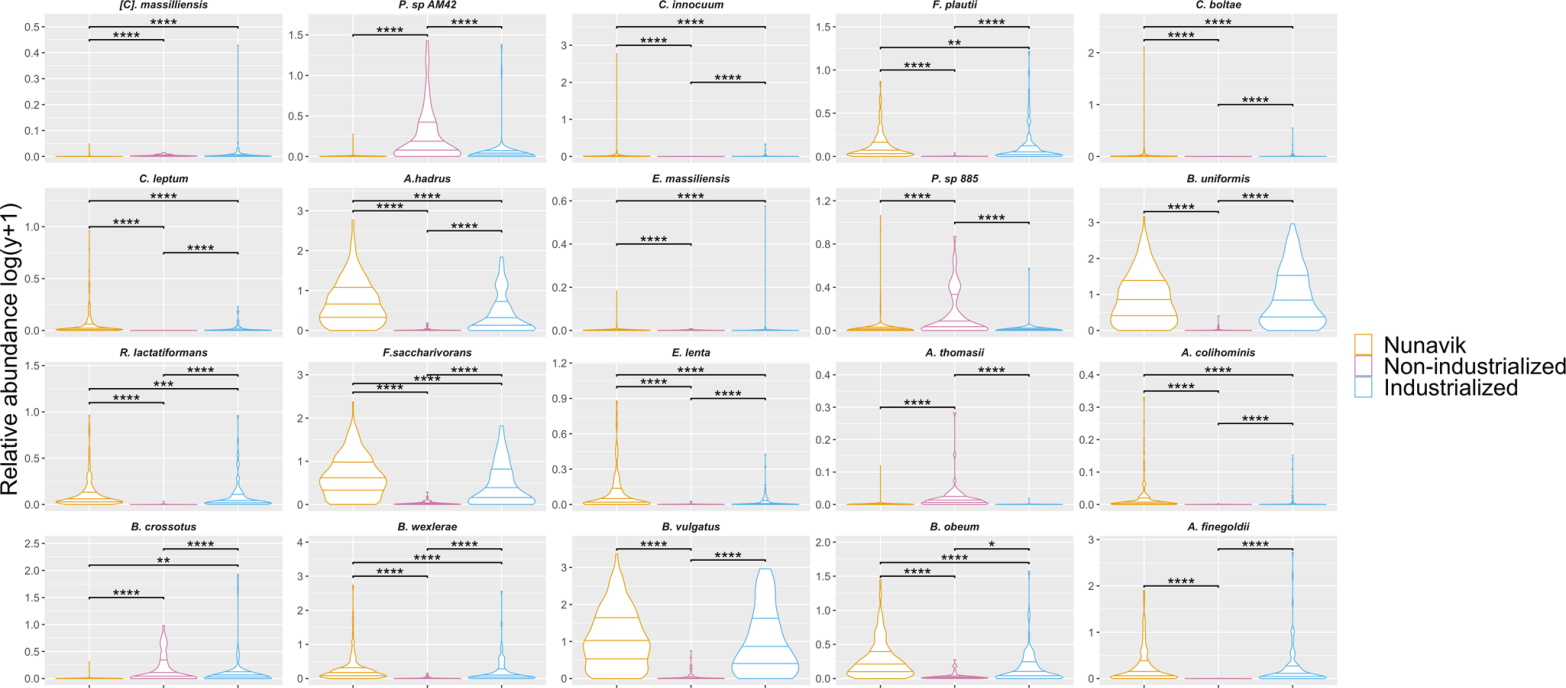
Top 20 Random Forest (RF) identified species that discriminate between Nunavik, non-industrialized and industrialized gut microbiome. Violin Plots represent the relative abundance (in copies per million, cpm) of RF identified species distinguishing between Nunavik and comparison microbiome (Wilcoxon-Mann-Whitney). (*p-value ≤ 0.05, **p-value ≤ 0.01, ***p-value ≤ 0.001, ****p-value ≤ 0.0001).

Using a random forest (RF) classifier, we specifically identified 20 discriminatory species that accurately separate the Nunavik gut microbiome from non-industrialized and industrialized counterparts (Table 1, Fig. 5) with a balanced accuracy score of 0.903. The segregation capability of the 20 signature species was estimated using PCoA on the Bray-Curtis dissimilarity matrix. Group separation on the PCoA1 axis accounts for 13.8% of the variance in dissimilarity (Supplementary Fig. 8).

**Table 1:** Most important species to discriminate Nunavik gut microbiome composition from comparison groups identified by random forest classifier, ranked based on median Gini importance.

Rank	Species	Median (and SD) Gini importance
1	[*Collinsella*] *massiliensis*	0.0246 ± 0.0042
2	*Prevotella sp AM42 24*	0.0229 ± 0.0058
3	*Clostridium innocuum*	0.0219 ± 0.0034
4	*Flavonifractor_plautii*	0.0191 ± 0.0044
5	*Clostridium_bolteae*	0.0174 ± 0.0047
6	*Clostridium_leptum*	0.0171 ± 0.0037
7	*Anaerostipes_hadrus*	0.0166 ± 0.0038
8	*Enorma_massiliensis*	0.0151 ± 0.0038
9	*Prevotella_sp_885*	0.0147 ± 0.0033
10	*Bacteroides_uniformis*	0.0140 ± 0.0029
11	*Ruthenibacterium_lactatiformans*	0.0135 ± 0.0030
12	*Fusicatenibacter_saccharivorans*	0.0135 ± 0.0031
13	*Eggerthella_lenta*	0.0131 ± 0.0025
14	*Anaerobiospirillum_thomasii*	0.0127 ± 0.0038
15	*Anaerotruncus_colihominis*	0.0123 ± 0.0030
16	*Butyrivibrio_crossotus*	0.0113 ± 0.0026
17	*Blautia_wexlerae*	0.0113 ± 0.0029
18	*Bacteroides vulgatus*	0.0112 ± 0.0026
19	*Blautia_obeum*	0.0104 ± 0.0023
20	*Alistipes_finegoldii*	0.0103 ± 0.0022

(*SD* standard deviation).

The bacterial signature of the Nunavik gut microbiome included a significantly higher median relative abundance of *C. innocuum*, *F. plautii*, *C. boltae*, *C. leptum*, *A. hadrus, B. uniformis, R. lactatiformans, F. saccharivorans, E. lenta*, *A. colihominis*, *B. wexlerae*, *B. vulgatus*, *B. obeum* and *A. finegoldii*. Some *E. lenta* strains (e.g., *E. lenta* DSM2243) possess the cardiac glycoside reductase (cgr) operon that degrades digoxin and reduces its bioavailibility^43^. Four detected signature species were also among the 20 most abundant (*A. hadrus*, *B. uniformis, F. saccharivorans, B. vulgatus*). Additionally, some have been studied for their positive effect on host health. *F. plautii* is under investigation for its regulatory effects on the immune system^44^. *A. colihominis*, is a major butyrate producer in the human colon, via its SCFA production, it could positively impact gut and brain health^45^. *Blautia spp*. are investigated for their potential probiotic role^46^.

### Nunavik gut microbiome functions

**Fig. 6:**
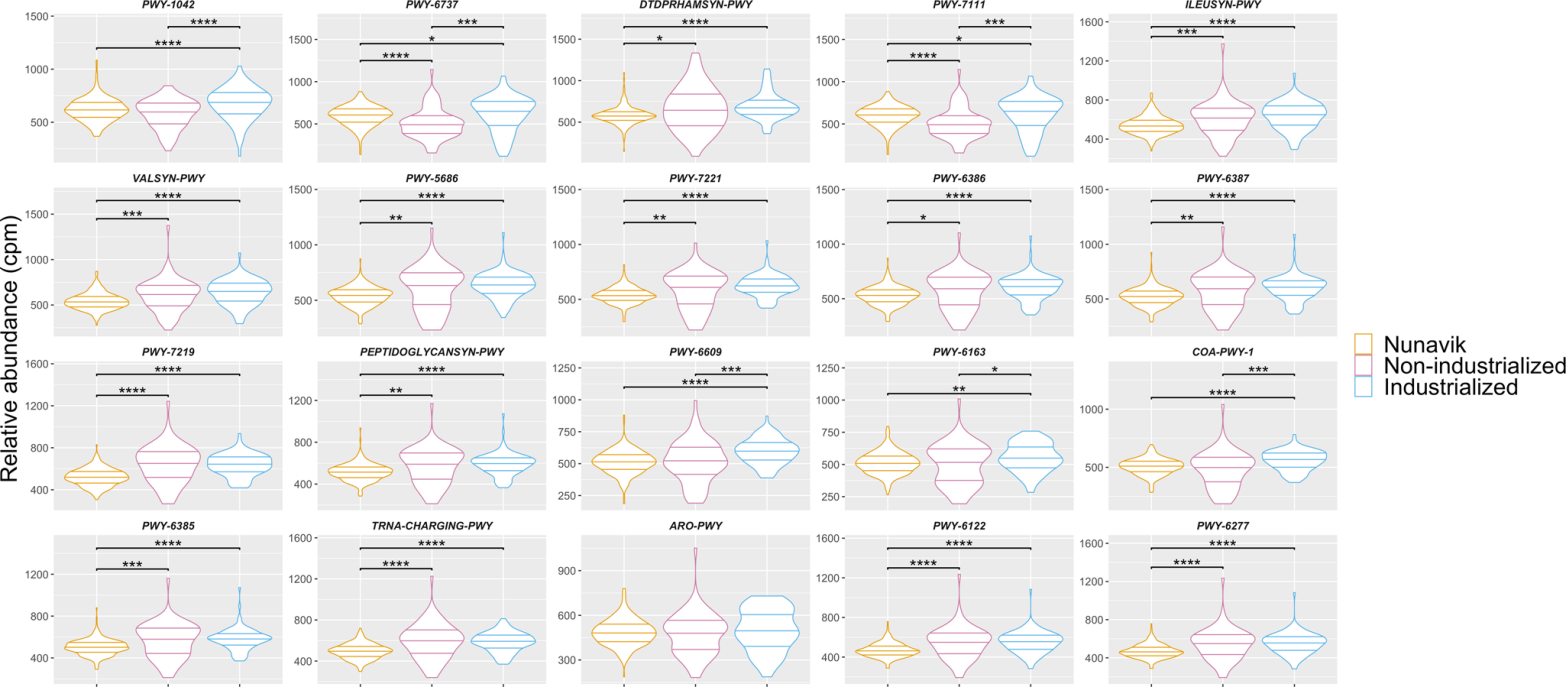
Top 20 most abundant pathways in the gut microbiome compared to individuals from non-industrial and industrial societies. Violin Plots represent the relative abundance (copies per million) of top 20 pathways in the Nunavik gut microbiome in comparison to non-industrialized and industrialized groups (Wilcoxon-Mann-Whitney; *: *p*-value ≤ 0.05, **p-value ≤ 0.01, ***: *p*-value ≤ 0.001, ****: *p*-value ≤ 0.0001). (PWY-1042 = PWY-1042: glycolysis IV (plant cytosol), PWY-6737 = PWY-6737: starch degradation V, DTDPRHAMSYN-PWY = DTDPRHAMSYN-PWY: dTDP-L-rhamnose biosynthesis I, PWY-7111 = PWY-7111: pyruvate fermentation to isobutanol (engineered), ILEUSYN-PWY = ILEUSYN-PWY: L-isoleucine biosynthesis I (from threonine), VALSYN-PWY = VALSYN-PWY: L-valine biosynthesis, PWY-5686 = PWY-5686: UMP biosynthesis, PWY-7221 = PWY-7221: guanosine ribonucleotides de novo biosynthesis, PWY-6386 = PWY-6386: UDP-N-acetylmuramoyl-pentapeptide biosynthesis II (lysine-containing), PWY-6387 = PWY-6387: UDP-N-acetylmuramoyl-pentapeptide biosynthesis II (meso-diaminopimelate containing), PWY-7219 = PWY-7219: adenosine ribonucleotides de novo biosynthesis, PEPTIDOGLYCANSYN-PWY = PEPTIDOGLYCANSYN-PWY: peptidoglycan biosynthesis I (meso-diaminopimelate containing), PWY-6609 = PWY-6609: adenine and adenosine salvage III, COA-PWY-1 = COA-PWY-1: coenzyme Abiosynthesis II (mammalian), PWY-6385 = PWY-6385: peptidoglycan biosynthesis III (mycobacteria), TRNA-CHARGING-PWY = TRNA-CHARGING-PWY: tRNA charging, ARO-PWY = ARO-PWY: chorismate biosynthesis I, PWY-6122 = PWY-6122: 5-aminoimidazole ribonucleotide biosynthesis I).

Two carbohydrates degradation pathways dominated the Nunavik gut microbiome (Fig. 6, Supplementary data 3). The pathway PWY-1042: glycolysis IV (plant cytosol) end product is pyruvate, which may cross-feed other species. Interestingly, *Bacteroides* spp. produce acetate and propionate from pyruvate, *Bifidobacterium* spp., *Ruminococcus* spp., *Prevotella* spp. use pyruvate to synthesize acetate, and *Anaerostipes* spp., *E. rectale*, *F. prausnitzii*, *Roseburia* spp. produce butyrate from pyruvate^29^.

PWY-6737: starch degradation V is a degradation pathway for ɑ-amylose and amylopectin, two resistant starches (RS), which are fermentable fibers. *R. bromii* and *B. adolescentis*, two of the top 20 species in the Nunavik gut microbiome, are starch degraders. Using RS, *R. bromii* produces acetate and sugars, while *B. adolescentis* synthesizes lactate, which cross-feeds other species. Starch degradation in the gut microbiome is beneficial for host health; it is associated with increased satiety, gut health, SCFAs production (particularly butyrate), decreased insulin resistance and reduced colorectal cancer risk^47^.

Although higher in comparison groups, ILEUSYN-PWY: L-isoleucine biosynthesis I (from threonine) and VALSYN-PWY: L-valine biosynthesis were two of the top pathways in Inuit metagenomes. L-isoleucine and L-valine are branched-chain amino acids (BCAA). Higher potential for BCAAs biosynthesis in the gut has been linked with liver cirrhosis and Type 2 Diabetes^48,49^. Instead, increased potential for inward transport of BCAAs appears to be protective^48^.

Two of the top 20 pathways were chorismate biosynthesis pathways, PWY-6163: chorismate biosynthesis from 3-dehydroquinate and ARO-PWY: chorismate biosynthesis I. Chorismate is a precursor of various bacterial products, such as phenylalanine, tryptophan, tyrosine and folates^50,51^. Tryptophan is the precursor of serotonin and its metabolism in gut bacteria, and the host is a central portion of the gut-brain axis research^51^.

**Fig. 7:**
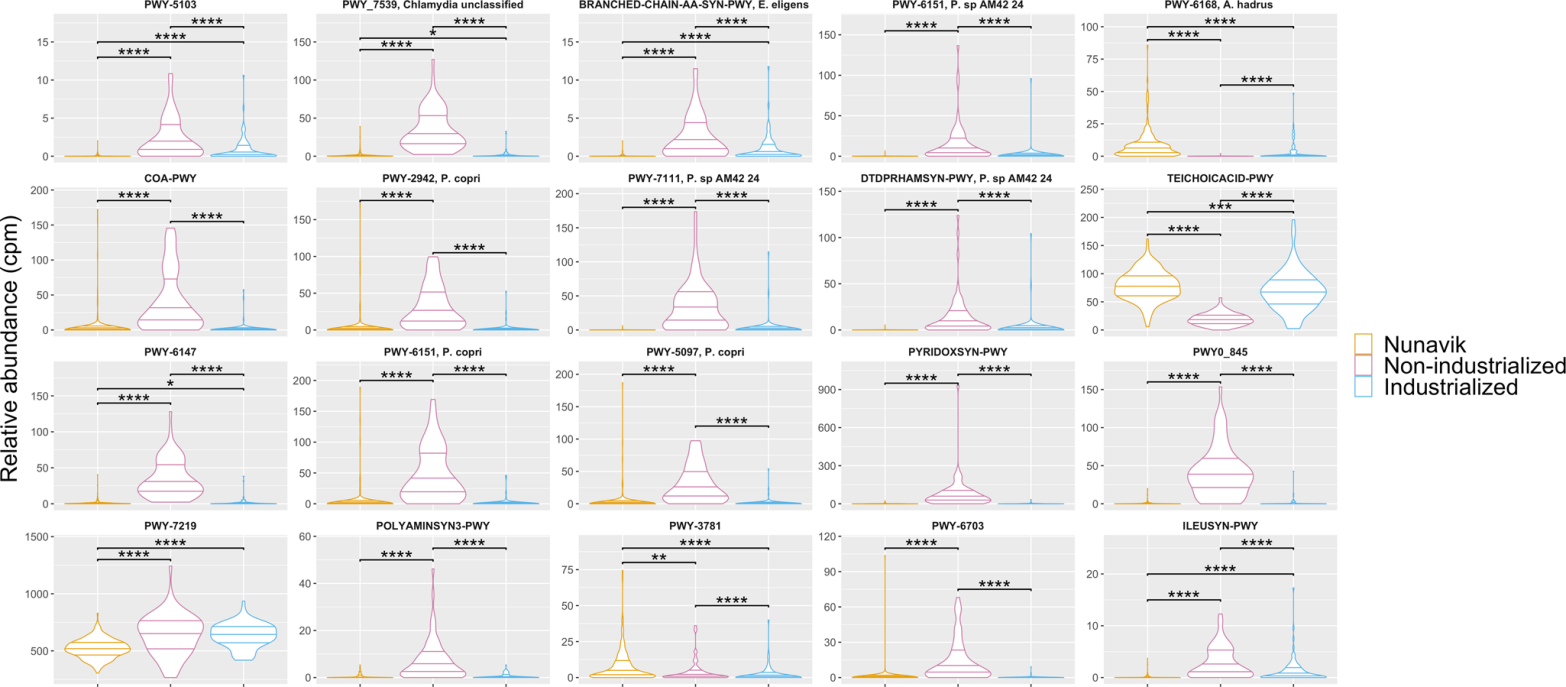
Top 20 Random Forest identified pathways that discriminate between Nunavik, non-industrialized and industrialized gut microbiome. Violin Plots represent the relative abundance (copies per million) of RF identified pathways distinguishing between Nunavik and comparison groups microbiome (in decreasing Gini importance order). (*p-value ≤ 0.05, **p-value ≤ 0.01, ***p-value ≤ 0.001, ****p-value ≤ 0.0001).

The RF classifier identified pathways accurately separating the Nunavik gut microbiome from non-industrialized and industrialized groups (Table 2, Fig. 7) with a balanced accuracy score of 0.903. Three BCAAs biosynthesis pathways were significantly reduced in Nunavik, (PWY-5103: L-isoleucine biosynthesis III | g__Eubacterium.s__Eubacterium_eligens, BRANCHED-CHAIN-AA-SYN-PWY: superpathway of branched amino acid biosynthesis|g_Eubacterium.s Eubacterium_eligens, and ILEUSYN-PWY: L-isoleucine biosynthesis I (from threonine)|g__Eubacterium.s__Eubacterium_eligens). Overall, the functional signature is consistent with taxonomic profiling.

**Table 2:** Most important pathways to discriminate Nunavik gut microbiome composition from comparison groups, identified by random forest, ranked based on median Gini importance.

Rank	Pathways	Median (and SD) Gini importance
1	PWY-5103: L-isoleucine biosynthesis III | g__Eubacterium.s__Eubacterium_eligens	0.0032 ± 0.0016
2	PWY-7539: 6-hydroxymethyl-dihydropterin diphosphate biosynthesis III (Chlamydia)|unclassified	0.0027 ± 0.0017
3	BRANCHED-CHAIN-AA-SYN-PWY: superpathway of branched amino acid biosynthesis|g_Eubacterium.s Eubacterium_eligens	0.0026 ± 0.0012
4	PWY-6151: S-adenosyl-L-methionine cycle I | g__Prevotella.s__Prevotella_sp_AM42_24	0.0025 ± 0.0016
5	PWY-6168: flavin biosynthesis III (fungi)|g__Anaerostipes.s__Anaerostipes_hadrus	0.0023 ± 0.0016
6	COA-PWY: coenzyme A biosynthesis I | g__Prevotella.s__Prevotella_copri	0.0023 ± 0.0015
7	PWY-2942: L-lysine biosynthesis III | g__Prevotella.s__Prevotella_copri	0.0023 ± 0.0015
8	PWY-7111: pyruvate fermentation to isobutanol (engineered)|g__Prevotella.s__Prevotella_sp_AM42_24	0.0022 ± 0.0015
9	DTDPRHAMSYN-PWY: dTDP-L-rhamnose biosynthesis I | g__Prevotella.s__Prevotella_sp_AM42_24	0.0022 ± 0.0020
10	TEICHOICACID-PWY: teichoic acid (poly-glycerol) biosynthesis	0.0022 ± 0.0017
11	PWY-6147: 6-hydroxymethyl-dihydropterin diphosphate biosynthesis I | unclassified	0.0021 ± 0.0011
12	PWY-6151: S-adenosyl-L-methionine cycle I | g__Prevotella.s__Prevotella_copri	0.0020 ± 0.0013
13	PWY-5097: L-lysine biosynthesis VI | g__Prevotella.s__Prevotella_copri	0.0020 ± 0.0016
14	PYRIDOXSYN-PWY: pyridoxal 5’-phosphate biosynthesis I | unclassified	0.0020 ± 0.0012
15	PWY0-845: superpathway of pyridoxal 5’-phosphate biosynthesis and salvage|unclassified	0.0020 ± 0.0013
16	PWY-7219: adenosine ribonucleotides de novo biosynthesis	0.0020 ± 0.0013
17	POLYAMSYN-PWY: superpathway of polyamine biosynthesis I | unclassified	0.0020 ± 0.0010
18	PWY-3781: aerobic respiration I (cytochrome c)	0.0020 ± 0.0015
19	PWY-6703: preQ0 biosynthesis|g__Prevotella.s__Prevotella_copri	0.0019 ± 0.0018
20	ILEUSYN-PWY: L-isoleucine biosynthesis I (from threonine)|g__Eubacterium.s__Eubacterium_eligens	0.0019 ± 0.0016

(*SD*: standard deviation).

## Discussion

The Nunavik Inuit are a unique population whose lifestyle, traditions and culture are profoundly shaped by their Arctic environment, factors impacting their dietary habits and the gut microbiome. In this study, we have generated the first in-depth description of the gut microbiome of young Nunavik Inuit and compared its microbial and functional structure to published shotgun metagenomics data from young, healthy individuals adhering to non-industrialized and industrialized lifestyles^7,9,10,13,22–24^. Taxonomic and functional profiling revealed that the gut microbiome of young Inuit exhibits greater diversity and is distinct and unique compared to non-industrialized and industrialized individuals selected from publicly available datasets.

Previous comparative metagenomics studies showed how lifestyle industrialization affects the human diet and lead to reduced intra-individual diversity of the gut microbiome. Low taxonomic diversity is consistently associated with diets low in fiber and high in saturated fats^2,5^. Nonetheless, recent findings suggest that the heterogeneity of food sources and quality of plant and animal foods shapes the gut microbiome composition and intra-individual diversity^52^. During the Q2017 health survey, data on the frequency of food intake was collected. It documents dietary diversity in Nunavik with weekly consumption of country and store-bought foods^21^. Plant-based foods alone are unlikely to drive the greater microbial diversity identified in Nunavik gut metagenomes.

Overall, our work conflicts with the previous study conducted with Inuit from Nunavut, another Inuit region of the Canadian Arctic. Girard et al. 2017^15^ reported that the gut microbiome of Nunavut Inuit and industrialized populations are similarly diverse and structured. These diverging results could be due to methodology differences, mainly amplicon sequencing, participants’ mean age, and the limited sample size in Girard and colleagues’ study. Additional studies should investigate Inuit dietary habits, which include traditional foods (nutrient dense with documented health benefits) and market foods (nutritious or non-nutritious) for their potential beneficial impact on the Nunavik gut microbiome and host’s health.

The Nunavik gut microbiome exhibits low variability, is homogenous and distinct from comparison groups. We identified that 6.5% of the variability among samples is attributable to lifestyle (Nunavik, non-industrialized or industrialized) (PERMANOVA; *R*^2^ = 0.06725, *p*-value = 0.001, Fig. 2). We also showed clear clustering of Nunavik metagenomes based on k-mer content and identified species/functions that separate Nunavik Inuit from comparison groups using RF classification. Nunavik samples harbor highly similar genomic contents, species, and function compositions. These similarities permit characterization and discrimination between Nunavik, non-industrialized, and industrialized metagenomes, suggesting shared lifestyle practices could shape the Nunavik gut microbiome. Besides dietary habits, multiple documented lifestyle factors (specific to Nunavik) may significantly explain the homogeneity of Nunavik metagenomes. Inuit food consumption and preparation practices are shaped by their Arctic environment. Meats, animal fat and fish can be consumed cooked, dried, frozen, raw, or fermented^21^. Other lifestyle factors such as food sharing and community living may be significant drivers^20,53^. A recent study of the Nunavik Inuit population’s genetic structure revealed two distinct genetic clusters among Nunavik Inuit^17^. Residents of two communities along Ungava Bay (Kuujjuaq and Kangiqsualujjuaq) were genetically different from communities near Hudson Bay. In contrast, our study of microbiome diversity and structure indicated that despite those genetic and geographical differences, the gut microbiome profiles of young Nunavik Inuit are comparable between both subregions (Supplementary Figs. 9, 10, and 11). Thus, host genetics does not appear to significantly shape the Nunavik gut microbiome, consistent with previous reports^52,54^.

Nunavik Inuit gut microbiome dominant species are SCFAs producing bacteria. SCFAs are primarily fermentation products of non-digestible fiber and protein that reach the large intestine^29,55^. Consistent with taxonomic composition, the resistant starch degradation V pathway was the second most prevalent in Nunavik samples. Resistant starch also emerged as one of the best fibers to stimulate butyrate production via species-cross feeding^47,56,57^. Nonetheless, as the Nunavik Inuit diet is rich in animal products, we cannot exclude that protein fermentation could also significantly drive community structure in the Nunavik gut microbiome. For example, the GLUTORN-PWY: L-ornithine biosynthesis pathway was detected, indicating glutamate degradation (53rd most prevalent pathway, Supplementary data 3). Additional work will be needed to study the effect of fiber and protein fermentation on species composition and their link to host health. Overall, our results suggest that the Nunavik gut microbiome taxonomic and functional signature features point to the adaptability of the gut microbiome^40,52^. This work also emphasizes the importance of saccharolytic species in the Inuit gut microbial community. Even if it is challenging to define a healthy or resilient microbiome, several characteristics found in the Nunavik gut microbiome, such as the high intra-individual diversity, the dominance of SCFA (primarily butyrate) producing species and the potential capability of the microbiota to recover from environmental stressors (e.g., antibiotics), all seem to be important factors related to reduced risks of chronic illness^40,52^.

We identified potential limitations to our study. First, we used comparison samples selected from external datasets^25^. Although we cannot entirely eliminate inter-study variability, it should be noted that the curatedMetagenomicData package is rigorously curated. Furthermore, we carefully selected comparison samples to match our methodology (sequencing depth, sequencing technology, age) to mitigate the impact of technical variability between groups. To remove non-biological sources of bias, we downloaded raw reads of selected comparison samples and processed them using the same pipeline used for Nunavik metagenomes. We show that the Nunavik gut microbiome exhibits higher microbial diversity than the non-industrialized and industrialized groups. We observed that sequencing depth and the number of reads passing quality control in Nunavik and Canadian samples were higher. We also found significant differences in the estimation of the total relative abundance of unknown species which could have an impact on intra-individual diversity analysis, However, the breakaway richness estimate was developed to characterize high diversity microbial data sets and incorporate unobserved taxa in its estimate^27^. Additionally, while strain-level analysis would be more informative, our species level characterization of the Inuit gut microbiome is a vast improvement over 16S rRNA sequencing, which is limited to genus-level resolution^58–60^. Lastly, dietary data were not included in the present study. Consequently, we can only consider the Q2017 health survey populational findings to identify lifestyle habits that potentially shape the Inuit gut microbiome. Our future work will explore associations between gut microbiome community structure and various lifestyle and sociocultural factors.

In summary, our study broadly describes the Nunavik youth gut microbiome. We have demonstrated that the Nunavik Inuit gut microbiome is diverse and unique. The community structure is consistent with Inuit lifestyle habits, such as their mixed-diet, traditional food preparation, and environment, as reported by the Q2017 health survey^21^. Intra-individual diversity, the dominance of SCFAs producing bacteria and the detection of keystone species for fast recovery post-antibiotic treatment suggest adaptability of the Nunavik gut microbiome ecosystem. Our study extends the scope of metagenomics profiling of the human gut microbiome and discusses results in the context of the unique Nunavik lifestyle and environment. In future work, we will investigate gut microbiome correlation with lifestyle and environmental factors of interest to Inuit communities. We anticipate that dietary habits and food consumption practices may significantly drive the homogeneity and uniqueness of the gut microbiome structure. Using metagenomics and metabolomics data combined with individual nutritional information from the Q2017 health survey, we expect to provide greater insight into the Nunavik Inuit adaptation to their way of life and environment.

## Methods

### Partnership with Nunavik organizations

The *Qanuilirpitaa* 2017? (In English: How are we now?, abbreviated Q2017) Nunavik Inuit Health Survey was made possible through a partnership between Université Laval, McGill University, and Trent University, the Nunavik Regional Board of Health and Social Services and the *Institut national de santé publique du Québec*. The Q2017 survey was designed and implemented in accordance with the principles of participatory research. The Data Management Committee of Q2017, composed of representatives of major Nunavik Inuit organizations and survey partners, approved the use of stool samples and access to the survey data for the present study. The Committee also discussed these findings with the researchers, reviewed the draft manuscript and provided comments. The health survey covered the population age group of 16 and over while the present study focuses on Nunavik youth (16–30-year-old) because they represent 44% of the population^61,62^.

### Participant enrollment and sample descriptions

Through the Q2017 health survey, 575 fecal samples were collected from August to September of 2017. Of these, 283 were donated by participants from the 14 Nunavik communities aged 16–30 years old (Supplementary Fig. 1). We successfully sequenced 279 using shotgun metagenomics at an average depth of 130.9 million reads per sample. Among those stool samples, 186 (66%) were obtained from females and 93 (33%) from male participants. Furthermore, 146 participants originated from Ungava Bay and 133 from Hudson Bay, and 275 participants declared being of Inuit descent while one was Caucasian and three selected other as ethnicity. Nunavik traditional foods (foods that are hunted, fished and gathered from the land: marine and terrestrial mammals, fish, shellfish, wild birds, and plants) play a key role in the Inuit culture and remain central to their diet. Nunavimmiut aged 16–29 reported in 2017 eating several country foods more frequently than older Nunavimmiut. Thus, for example, weekly consumption of traditional foods was reported by many of them: beluga meat (72%), seal meat (52%), caribou (97%), goose (81%), wild bird eggs (51%), shellfish (56%), wild berries (86%), and suuvalik or uarutilik (72%)^21^. Population of Nunavik face elevated blood levels of mercury (due to exposure through consumption of certain traditional foods), lead (due probably to use of firearms fired with lead ammunition or to exposure during the firearms cleaning), and cadmium (associated with cigarette smoking and second-hand smoke), markedly higher than in the general Canadian population. Regarding the 16–29 cohort specifically, blood levels were lower among this group when compared to older Nunavimmiut for mercury and lead, but higher for cadmium^63^. Tobacco smoke is widespread in Nunavik in general and among Nunavimmiut aged 16–30 years particularly: 70.2% and 77.9% of those aged 16–20 years and 21–30 years, respectively reported daily smoking in 2017. Binge drinking (5 drinks or more on one occasion) in the year preceding the survey was reported by 75.4% and 81.2% of Nunavimmiut between 16–20 and 21–30, respectively^64^.

### Sample collection

The Q2017 survey was conducted onboard the Canadian research icebreaker CCGS *Amundsen*. Within 24 h of their visit on the ship, participants directly deposited their stools in a stool collection container (Commode Specimen Collection System, Biomedical Polymers, Inc., Gardner, MA, USA) and stored them in their refrigerator. They brought the sample with them during their visit to the CCGS *Amundsen*, where they transferred their samples to the personnel of the Q2017 survey. On the ship, samples were stored in a cold room at 4 °C, then aliquoted into two 2 mL tubes. The first aliquot was pure stool, and the second was stool mixed with 10% glycerol. Finally, aliquots were frozen at -80 °C. This study was approved by the research ethics committee of the *Centre Hospitalier Universitaire de Québec* (CHUQ), committee number FWA00000329 and IRB00001242. The study IRB approval # is MP-20-2019-4511. The staff of the *Qanuilirpitaa?* 2017 (Q2017) Nunavik Inuit Health Survey collected the informed consents from all participants.

### Comparison groups selection

The comparison groups were established by selecting data from the curatedMetagenomicData R package^25^. This package is a collection of high quality curated per-sample metadata with accession number to raw reads. Sample selection was based on sequencing depth (reads per samples >40 million), sequencing technology (Illumina), age (16–30), lifestyle (non-industrial vs industrial), and only metagenomes from healthy individuals were considered for analysis. In total, 177 comparison samples were utilized, of which 73 non-industrialized^7,9,13^ (average of 90.5 million reads per sample), 104 industrialized^10,22–24^ (average of 64.1 million reads per sample), 88 were donated by females and 89 by males.

### DNA extraction, library preparation, and sequencing

All pure stool samples were thawed and transferred from 2 mL tubes to 96-well plates (PowerBead DNA Plates, Qiagen) and then sent to McGill University and Génome Québec Innovation Centre (Montreal, Quebec, Canada) for DNA extraction. DNA extraction was performed using QIAamp 96 PowerFecal QIAcube HT Kit and the Qiacube HT instrument from Qiagen (Qiagen). gDNA was quantified using the Quant-iT™ PicoGreen® dsDNA Assay Kit (Life Technologies). A total of 279 samples yielded sufficient gDNA. Libraries were generated using the NEBNext Ultra II DNA Library Prep Kit for Illumina (New England BioLabs) per the manufacturer’s recommendations. Adapters and PCR primers were purchased from IDT (Integrated DNA Technologies, Inc., Iowa, USA). Size selection of libraries containing the desired insert size was performed using SparQ beads (Qiagen). Libraries were quantified using the Kapa Illumina GA with Revised Primers-SYBR Fast Universal kit (Kapa Biosystems). The average size fragment was determined using a LabChip GX (PerkinElmer) instrument. Paired-end sequencing (2 × 151 bp) was performed using NovaSeq 6000 (Illumina). Sequencing was done in four runs, with an average QC score = 35.80 (minimum 33.41–maximum 36.25 of the mean per base sequence quality) and an average of 130.9 million (±24.2 million standard deviation) reads per sample obtained. Finally, we used two internal controls to assess possible contamination of the used extraction kit and found no significant amount of contaminant DNA.

### Quality assessment

YAMP (Yet Another Metagenomic Pipeline) was used to perform a quality assessment on shotgun sequencing results^65^. Raw reads from the Nunavik and comparison samples were processed by first deduplicating reads (discarding identical reads). Then reads were filtered to remove adapters and artefacts. The final decontamination step is the removal of reads that do not belong to a gut microbiome (e.g: human reads, plant reads). In the Nunavik samples, a median of 39,930 reads ([5,944–1,107,985] 90% confidence interval) mapped to the human genome. This resulted in an average of 88.9% (±4.6% std) read survival to all quality control steps. In non-industrialized controls, a median of 10 reads ([0–858] 90% confidence interval) mapped to the human genome. The average read survival to all quality control steps was 88.4% (±8.7% std). A median of 702 reads ([2–24,967] 90% confidence interval) mapped to the human genome in industrialized metagenomes and the average read survival to quality control steps was 85.7% (±7.5% std). All subsequent analyses made use of the cleaned sequence files produced by YAMP.

### Taxonomic and functional profiling and genomic content comparison

Metagenomic profiling (taxonomic) was performed using MetaPhlAn 3.0. We used ChocoPhlAn (mpa_v30_CHOCOPhlAn_201901_marker_info) as database of reference marker genes using default parameters^26^. Functional profiling was conducted with HUMAnN 3.0 software, with uniref90_201901 and ChocoPhlAn (v296_201901) as reference databases. Assembly of reads into contigs was done using MEGAHIT software, with kmer sizes of 21, 29, 31, 39, 59, 79, 99, 119, and 141^66^. Quality assessment of assemblies was conducted with MetaQUAST^67^. Ray Surveyor was used to compare k-mer content between samples (k-mer = 31)^28^. Only MEGAHIT assembled contigs ≥500 nucleotides in length were considered for Ray Surveyor analysis. All bioinformatic tools were launched on Calcul Québec and Compute Canada servers, and subsequent analyses were conducted either in Python notebooks (V3) or in RStudio.

### Statistics and reproducibility

We used the breakaway^27^ R package (v4.7.3) to estimate species richness using the breakaway richness estimate and the vegan package for Simpson, Shannon-Weiner, and Inverse-Simpson indices computation^68^. The global statistical significance of distribution difference among the three groups was tested by applying Kruskal–Wallis test followed by Shapiro & Wilk test to assess per-group distribution normality. When the data followed a normal distribution we used T-test to establish the significance of difference among each pair of groups. When the distribution was not normal, we used the Wilcoxon-Mann-Whitney test. Beta-diversity analyses were performed using the vegan Community Ecology Package for R (v2.5-7)^68^. We used Principal Coordinates Analysis and ggplot2 (v3.3.3) to visualize results in RStudio. Permutational Multivariate Analysis of Variance using distance matrices (adonis2 function) and ANalysis Of Similarities (anosim function) were computed with the vegan package. All analyses performed to assess statistical significance were done for Nunavik samples *n* = 279, non-industrialized samples *n* = 73, and industrialized samples *n* = 104.

### Random forest classification

The RF algorithm builds models composed of an ensemble of decision trees^69^. Random forest models predict the class of some examples based on the values of their features. During the training phase, decision trees are built on a sub-part of the training data. The prediction of the ensemble model is the majority class in the predictions of the decision trees that compose the model. In our case, the decision trees are made with the CART algorithm, which uses the Gini impurity metrics. The importance of a feature defined at a node with a threshold value is the quality of the split made by this node according to the metrics (Gini impurity decrease). The importance of a feature in the RF model is defined as the normalized total importance of nodes that rely on this feature (Gini importance). The most important feature is the most useful at predicting the class of the examples. We used the random forest algorithm implemented in the scikit-learn (v0.24) Python package^70^. The CART decision trees construction algorithm defined two multi-class supervised learning problems, relying on the same method. In the first, resp. second, problem, the data matrix contains the abundance of species, resp. functions, (features) in each sample (examples) and the classes to predict are the lifestyle of the samples. Samples were randomly split into a train set and a test set, with 70% of examples for training and the remaining 30% for testing. This operation was repeated 100 times (i.e., 100 splits) with different random seeds. The random forest algorithm was applied on each training set, giving 100 models. For each of the 100 splits, the hyperparameters were selected using only the training part of the split. Hyperparameters tuning was done with 5-fold cross-validation on training data. For each split, one model was selected and fitted on training data and applied to predict labels of the testing data. The hyperparameter (number of trees in the forest) value selected was 100 on 32 of the splits, and 1000 on the 68 other splits for the predictive models based on species. For the prediction with functions, the values of the hyperparameter were 100 on 35 of the splits and 1000 on the other 65 splits. Performance prediction of models was evaluated on their respective test sets using accuracy and F1 scores. The reported performance is the average of the 100 scores from the predictions made on testing data. For each model, we computed the importance of the features based on the decrease in Gini impurity criterion. We ranked the features given their median importance score over the 100 models and selected the 20 most relevant.

### Reporting summary

Further information on research design is available in the Nature Portfolio Reporting Summary linked to this article.

## Supplementary information

Supplementary Information

Description of Additional Supplementary Data

Supplementary Data 1

Supplementary Data 2

Supplementary Data 3

Reporting summary

## Data Availability

The data that support the findings of this study were used under copyright agreement and so are not publicly available without a request. In accordance with the First Nations principles of ownership, control, access, and possession (OCAP®), the Nunavik Regional Board of Health and Social Services is the owner of the data and biological samples collected during the *Qanuilirpitaa? 2017* health survey on behalf of the Inuit population of Nunavik. Any request for data access should be addressed to the *Qanuilirpitaa? 2017* Data Management Committee (email: nunavikhealthsurvey@ssss.gouv.qc.ca) that oversees the management of the survey data and biological samples. Additional information on data ownership, management, and access, as well as the Data/Biological Samples Request & Analysis Proposal Application Form are available in the Methodological report of the *Qanuilirpitaa*? 2017 (see Appendix ‘Policy on the management of databases and biological samples’, Section 7, pages 14–16 (415–417) and Appendix B, pages 23–27 (424–428); https://nrbhss.ca/sites/default/files/health_surveys/A11991_RESI_Rapport_methodologique_EP4.pdf). The 16–30 cohort is thoroughly described in socio-demographic terms (gender, marital status, education, employment, income, etc.) in the sociodemographic characteristics report https://nrbhss.ca/sites/default/files/health_surveys/A12468_RESI_Sociodemographic_Characteristics_EP4.pdf. Populational dietary information is available in the country and market food consumption and nutritional status thematic report. Information on the frequency of traditional activities (hunting, fishing, harvesting, and berry picking) is available in the report on the sociocultural determinants of health; section 4.2.2, page 10. Information on exposure to contaminants can be found in the two reports on this Environmental Contaminants: Metals and Environmental Contaminants: Persistent Organic Pollutants and Contaminants of Emerging Arctic Concern. Exposure to alcohol, tobacco, and other substances has also been documented in the report on substance use.

## Code availability

The code for RF analysis, statistical analysis, and visualization are available from the GitHub repository (https://github.com/corbeillab/GutMetagenomeOfTheNunavikYouth).
