# Supplementary material for: Gut metagenome profile of the Nunavik Inuit youth is distinct from industrial and non-industrial counterparts
Source: Commun Biol. 2022 Dec 24;5:1415. doi: 10.1038/s42003-022-04372-y (PMC9790006; doi:10.1038/s42003-022-04372-y)
Supplement: Supplementary file 1 — Supplementary Information [file 42003_2022_4372_MOESM1_ESM.pdf]

Supplementary materials: Gut metagenome profile of the Nunavik Inuit youth

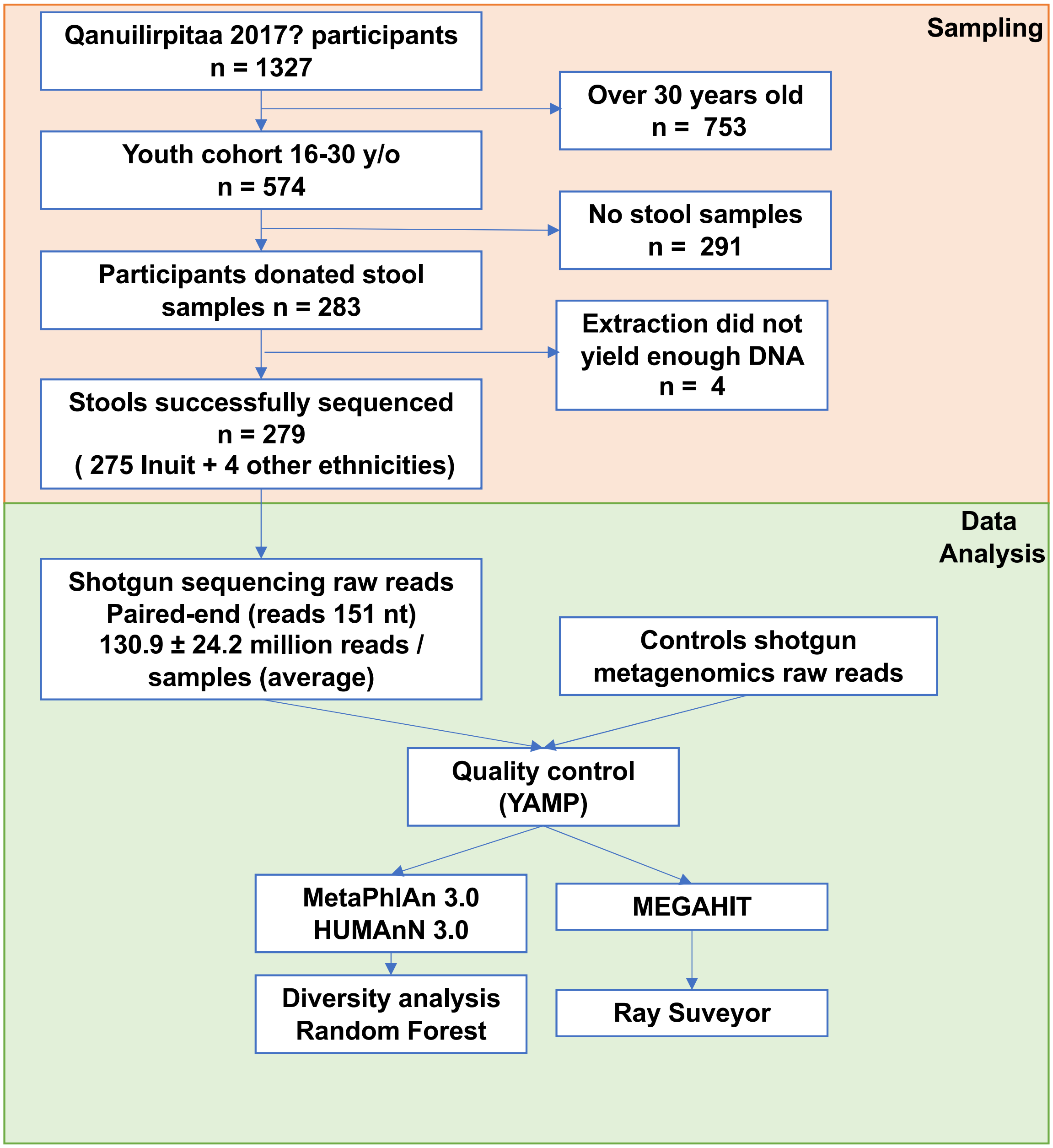

Supplementary figure 1: Sampling and data analysis workflow

	Phyla	Class	Order	Families	Genera	Species	Pathways
**Combined data**	15	29	47	92	228	714	26046
**Nunavik**	15	26	39	71	189	561	20304
**Non-industrialized**	12	25	42	78	164	451	15165
**Industrialized**	14	24	35	61	149	445	14521

Supplementary table 1: Number of taxa detected in combined data, in Nunavik, non-industrialized and industrialized groups data.

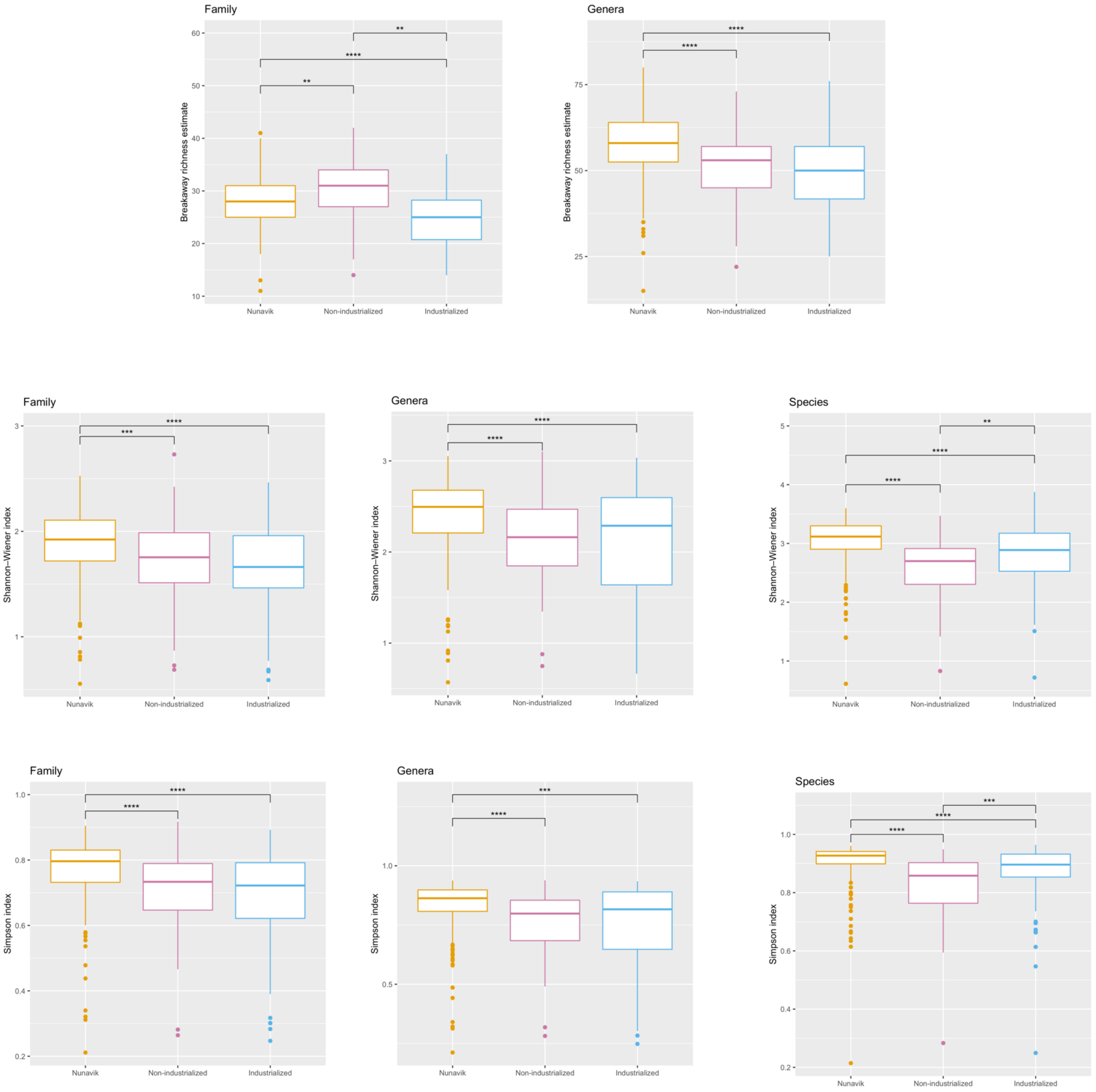

Supplementary figure 2: Alpha diversity, using different diversity metrics. Breakaway richness (T-test), Shannon-Weiner (Wilcoxon-Mann-Whitney test), and Simpson (Wilcoxon-Mann-Whitney test) highlight a higher alpha-diversity in the Nunavik gut microbiome, at species, genus and family levels. (*: p-value ≤ 0.05, **: p-value ≤ 0.01, ***: p-value ≤ 0.001, ****: p-value ≤ 0.0001)

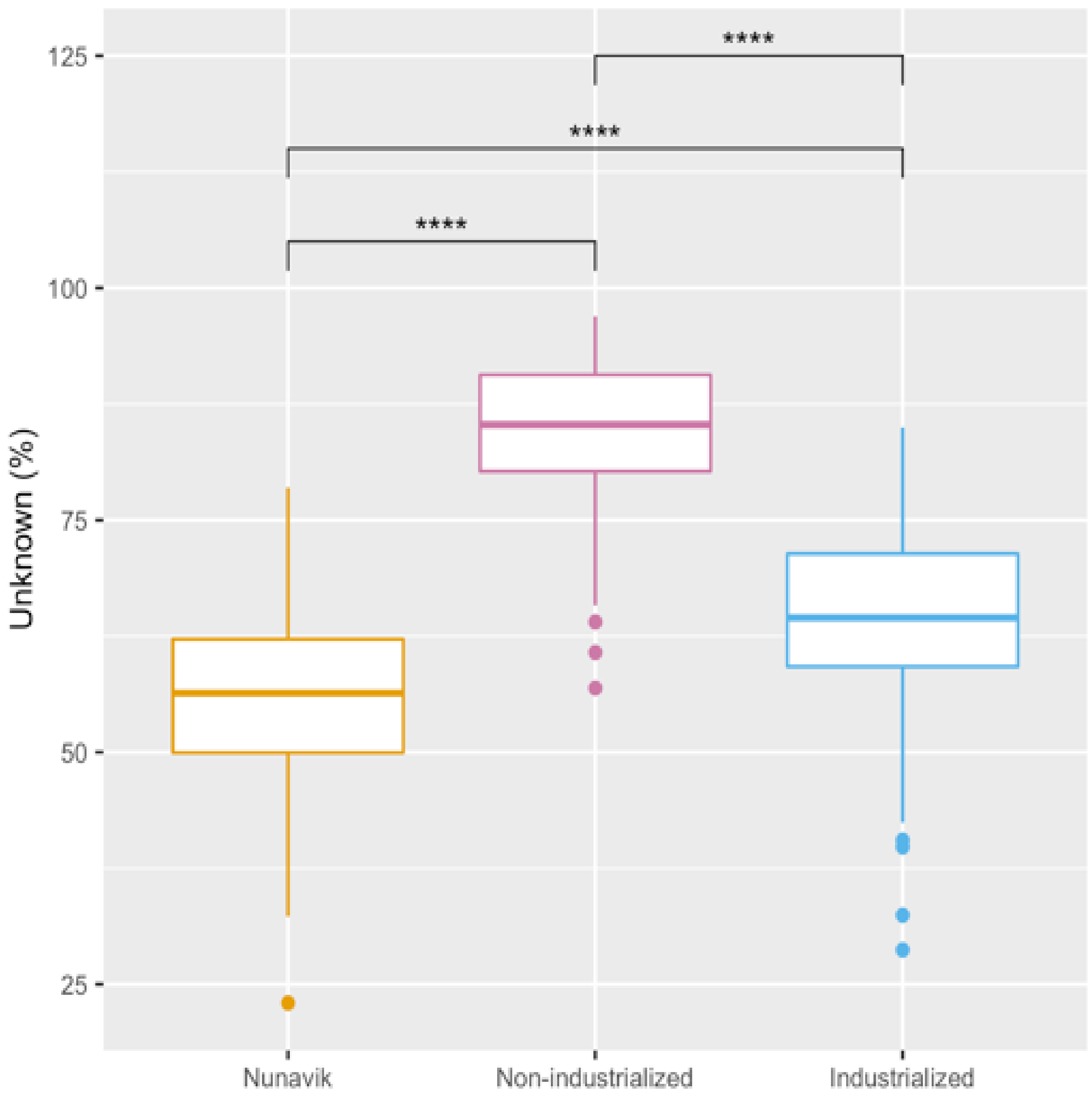

Supplementary figure 3: MetaPhlAn 3.0 estimation of the unknown percentage of taxa in each Nunavik, non-industrialized and industrialized metagenomes (Wilcoxon-Mann-Whitney test). (*: p-value ≤ 0.05, **: p-value ≤ 0.01, ***: p-value ≤ 0.001, ****: p-value ≤ 0.0001)

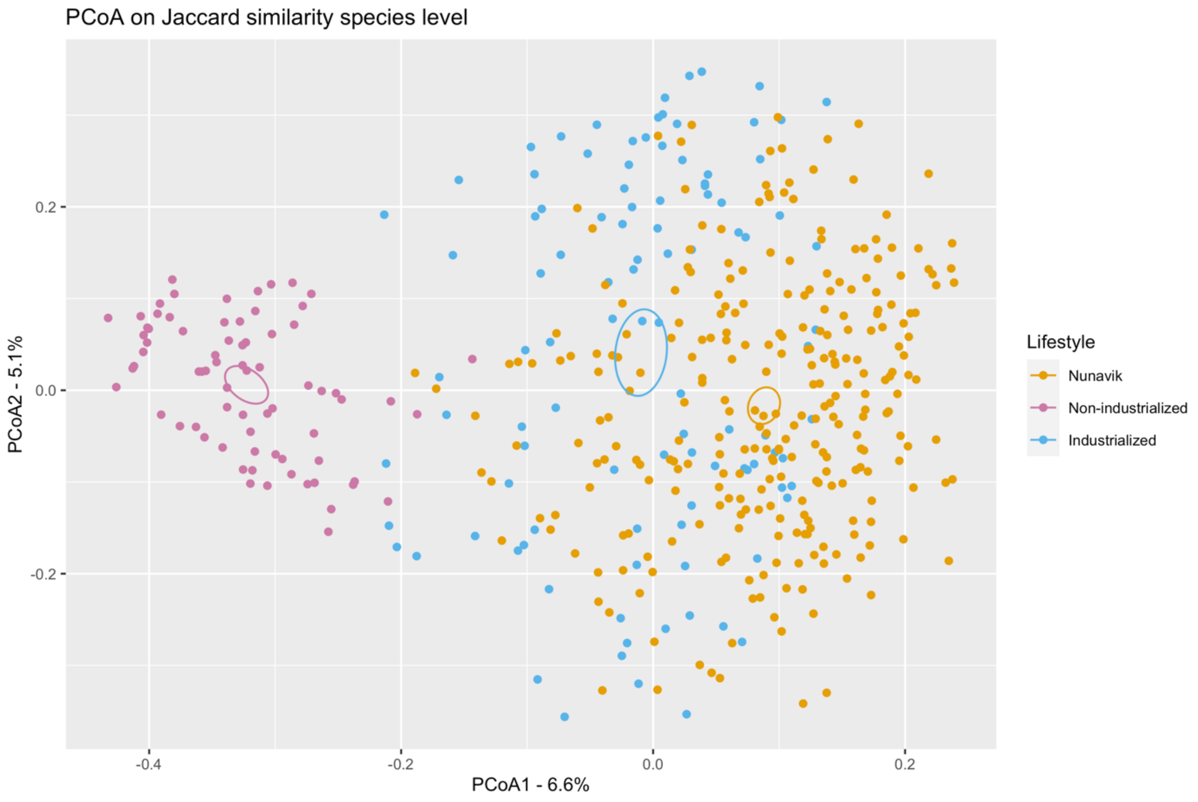

Supplementary figure 4: Jaccard similarity of the microbiome in Nunavik Inuit and in individuals from non-industrial and industrial societies. PCoA on Jaccard similarity shows significant separation between species composition from Nunavik, compared to non-industrial (n = 73) and industrial (n = 104) populations. Ellipses represent the barycenter of the sample groups with their 95% confidence interval. PERMANOVA was used to evaluate significance of lifestyle (R^2 = 0.06725, p-value = 0.001). Age and sex had a minor effect on the observed variance (age; R^2 = 0.00454, p-value = 0.001, sex; R^2 = 0.00457, p-value = 0.001). Analysis of Similarities (ANOSIM) demonstrated significant differences between groups based on lifestyle (lifestyle, R = 0.52, p-value = 0.001; age, R = 0.008, p-value = 0.195; sex, R = 0.028, p-value = 0.01).

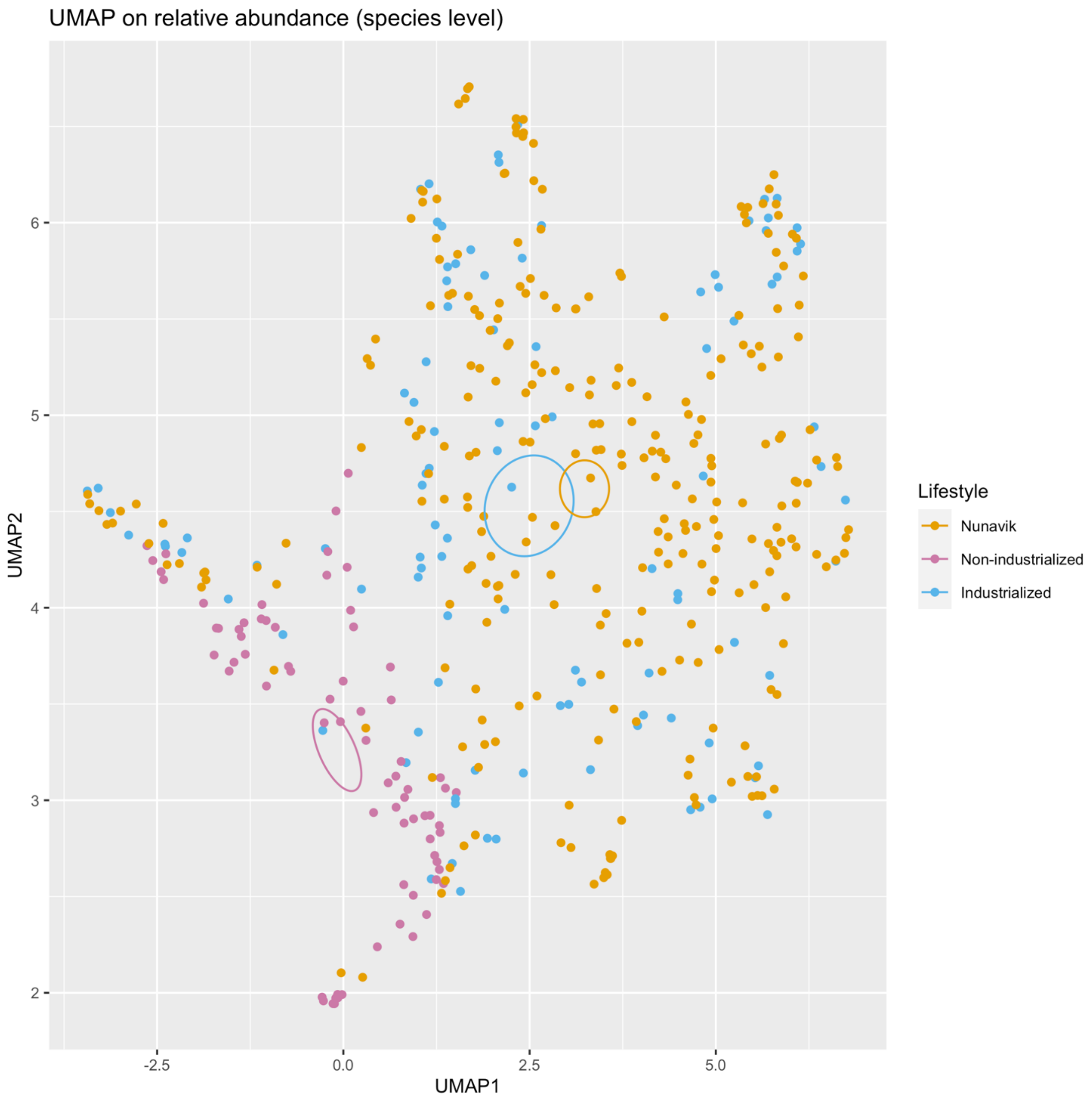

Supplementary figure 5: UMAP representation of the microbiome in Nunavik Inuit and in individuals from non-industrial and industrial societies. UMAP on relative abundance shows significant separation between species composition from Nunavik, compared to non-industrial (n = 73) and industrial (n = 104) populations. Ellipses represent the barycenter of the sample groups with their 95% confidence interval.

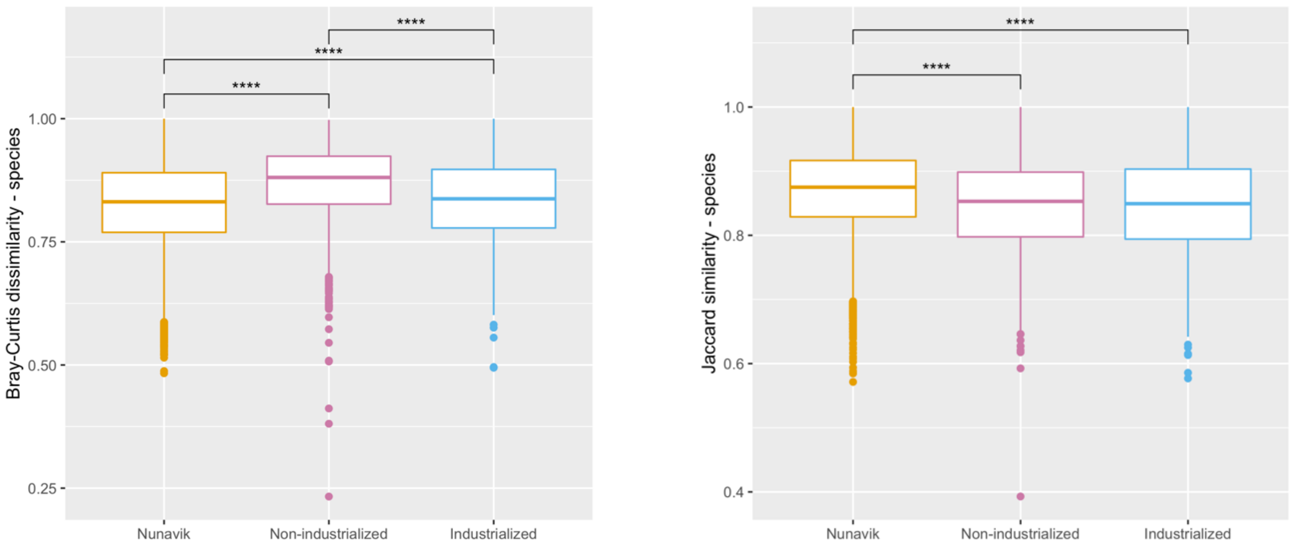

Supplementary Figure 6: Beta-diversity is lower in Nunavik compared to comparison groups using Bray-Curtis dissimilarity and Jaccard similarity. (Wilcoxon-Mann-Whitney test). (*: p-value ≤ 0.05, **: p-value ≤ 0.01, ***: p-value ≤ 0.001, ****: p-value ≤ 0.0001)

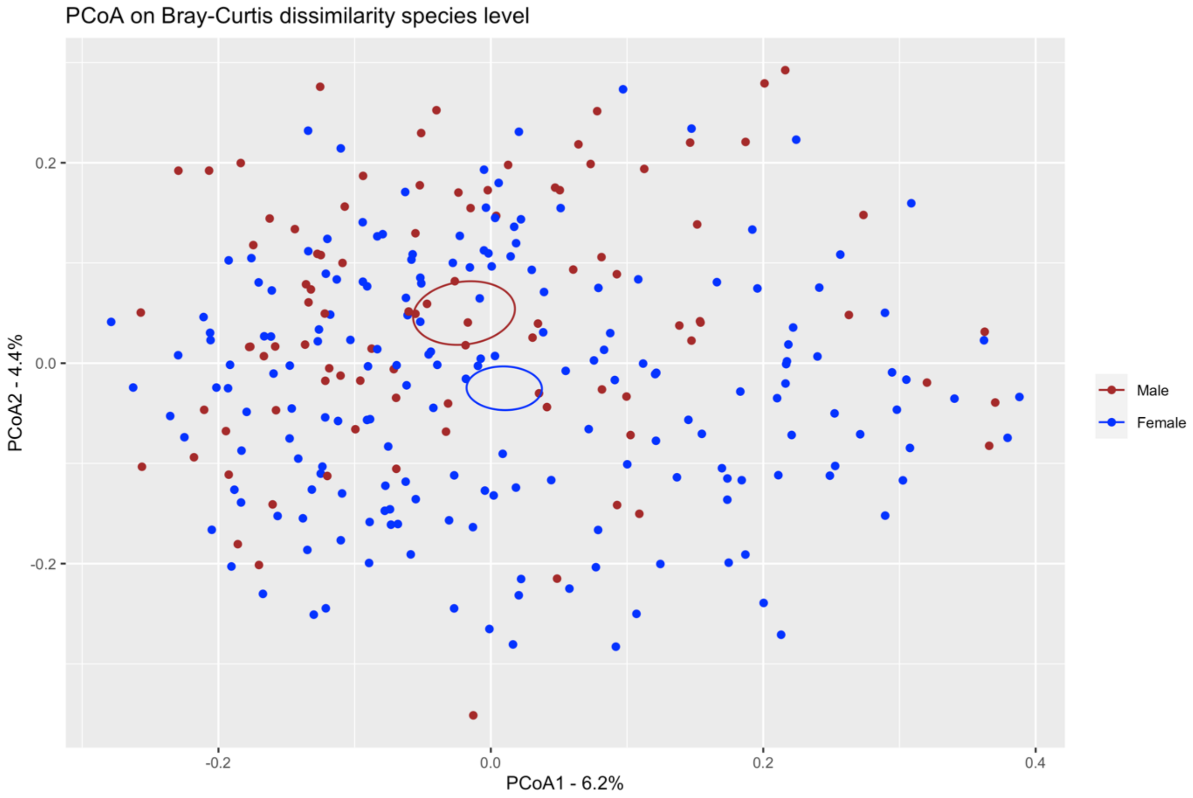

Supplementary figure 7: PCoA on Bray-Curtis dissimilarity shows minor variance between female and male participants in Nunavik (PERMANOVA: R^2 = 0.00774, p-value = 0.001).

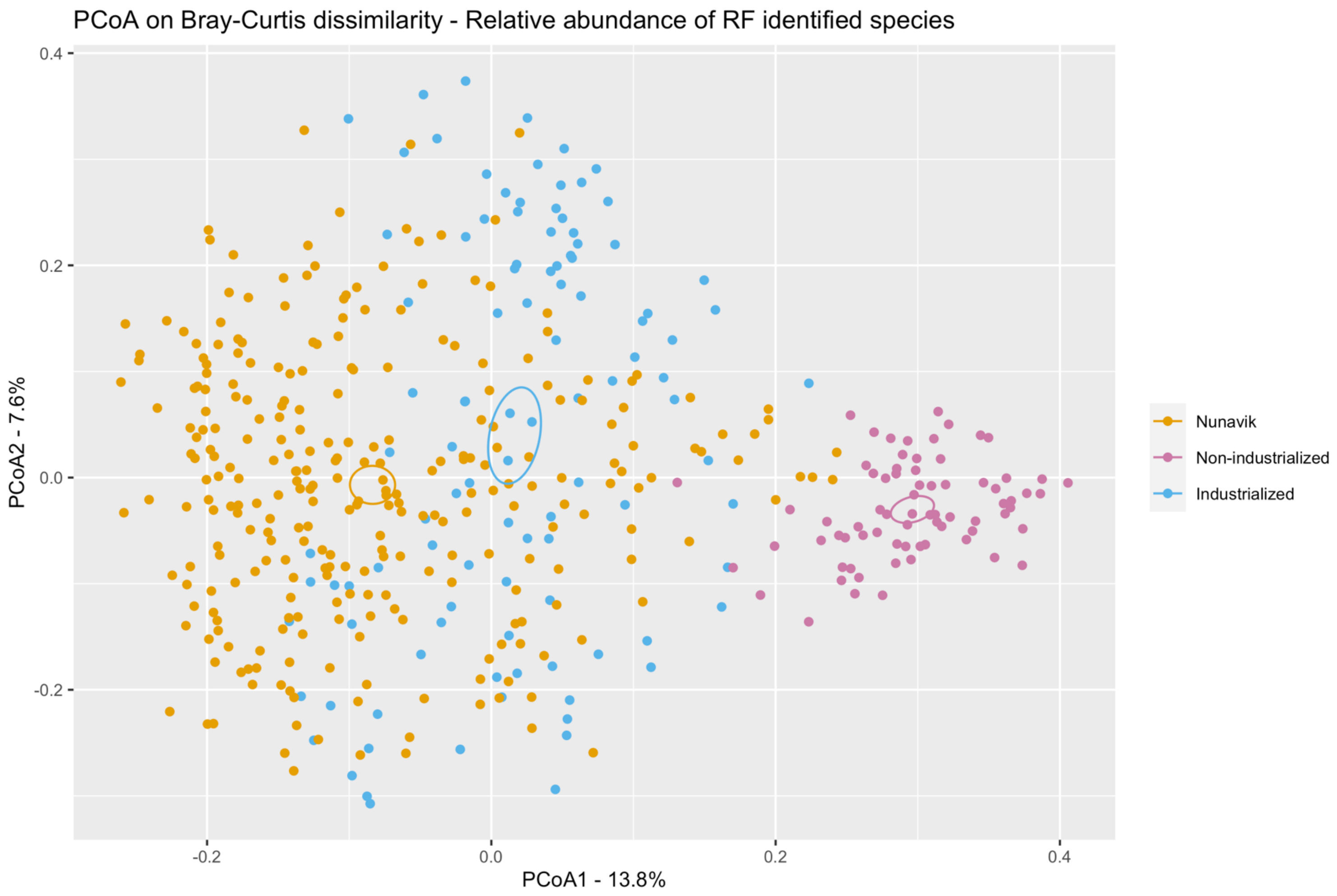

Supplementary figure 8: Random Forest identified species relative abundance significantly separate Nunavik samples from comparison groups. PCoA on Bray-Curtis dissimilarity shows significant separation between groups of Nunavik, Non-industrial (n = 73) and Industrial (n = 104) populations. Ellipses represent the barycentre of the sample groups with 95% confidence interval. PERMANOVA was used to evaluate significance of lifestyle (R^2 = 0.19041, p-value = 0.001). Age and sex had a minor effect on the observed variance (age; R^2 = 0.00359, p-value = 0.028, sex; R^2 = 0.00537, p-value = 0.002). Analysis of Similarities (ANOSIM) demonstrated significant differences between groups based on lifestyle (lifestyle, R = 0.5656, p-value = 0.001; age, R = -0.0103, p-value = 0.847, sex; R = 0.01402, p-value = 0.118). (*: p-value ≤ 0.05, **: p-value ≤ 0.01, ***: p-value ≤ 0.001, ****: p-value ≤ 0.0001)

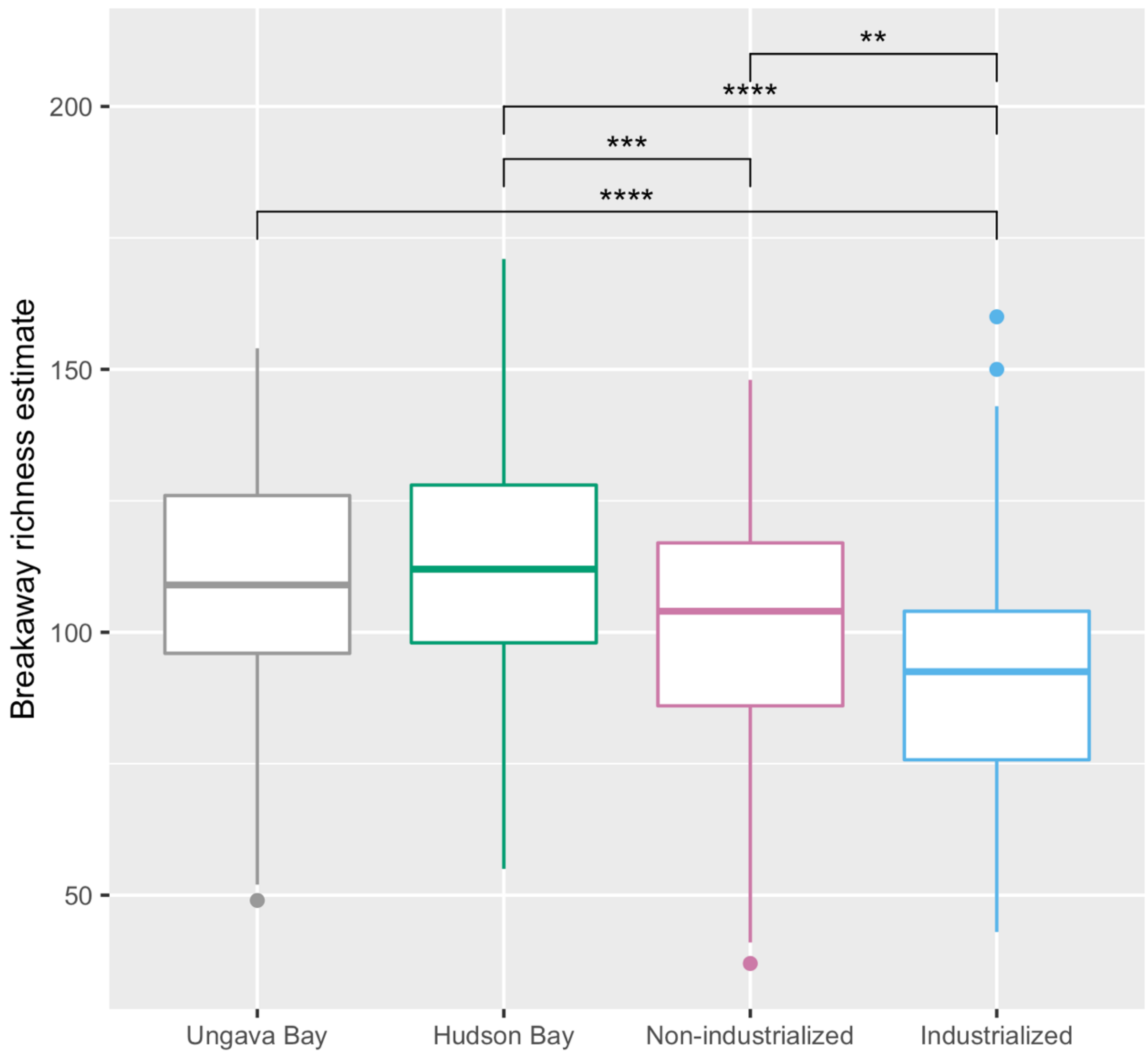

Supplementary figure 9: Alpha-diversity comparison between participants from the Nunavik Ungava and Hudson Bay. Breakaway richness estimate did not show differences in levels between participants from Ungava and Hudson Bay. (T-test, *: p-value ≤ 0.05, **: p-value ≤ 0.01, ***: p-value ≤ 0.001, ****: p-value ≤ 0.0001)

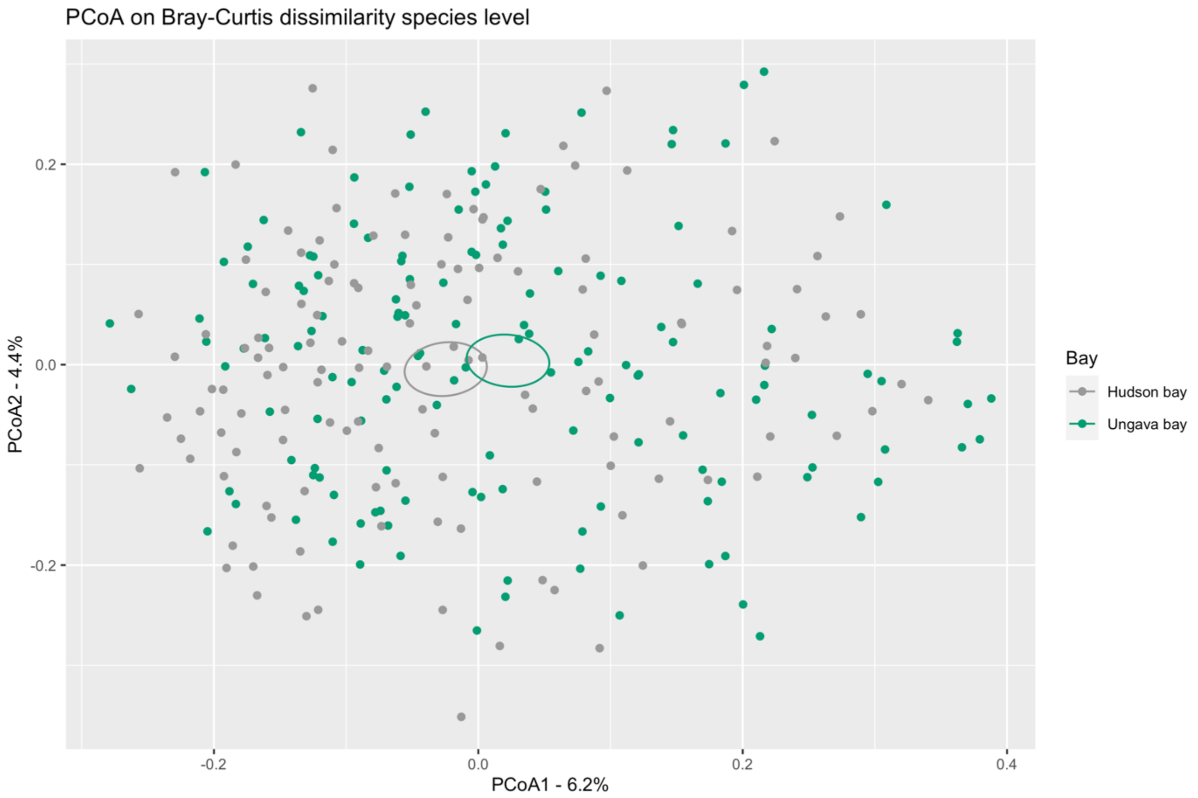

Supplementary figure 10: PCoA on Bray-Curtis dissimilarity shows minor variance between participants from Ungava and Hudson Bay (R^2 = 0.0079, p-value = 0.001). Age and sex had also had a minor effect on the observed variance (age; R^2 = 0.00802 p-value = 0.001, sex; R^2 = 0.00774, p-value = 0.001).

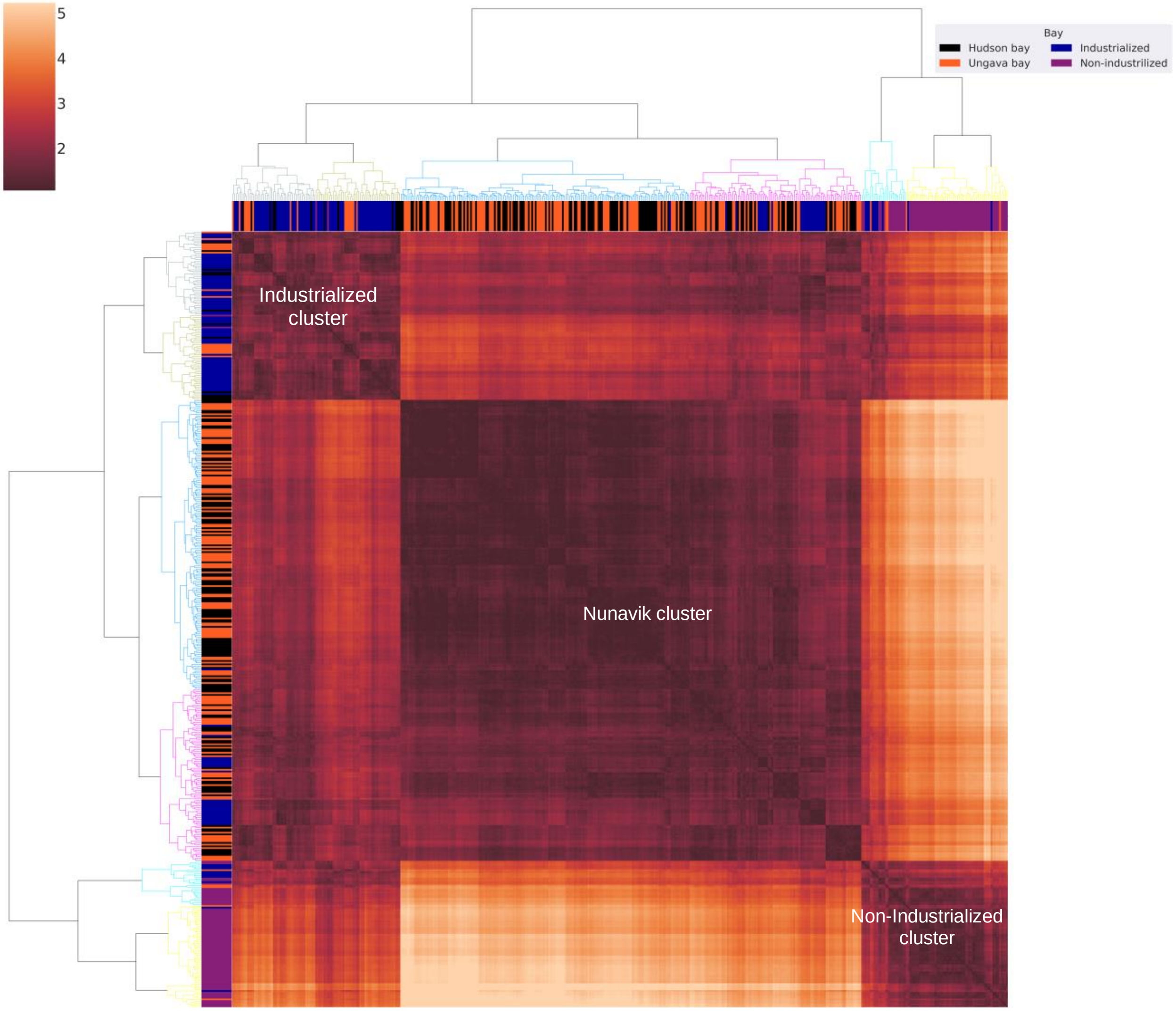

Supplementary figure 11: Heatmap depicting the Euclidean distance between genomic content of Ungava and Hudson gut microbiome compared to non-industrialized and industrialized populations. It did not identify differences between participants from Ungava and Hudson Bay.
