# Supplementary material for: Gut metagenome profile of the Nunavik Inuit youth is distinct from industrial and non-industrial counterparts
Source: Commun Biol. 2022 Dec 24;5:1415. doi: 10.1038/s42003-022-04372-y (PMC9790006; doi:10.1038/s42003-022-04372-y)
Supplement: Supplementary file 2 — Description of Additional Supplementary Data [file 42003_2022_4372_MOESM2_ESM.docx]

**Description of Additional Supplementary Files**

**File name:** Supplementary Data 1

**Description:** The source data behind the graphs representing the top 20 species and top 20 pathways

**File name:** Supplementary Data 2

**Description:** Modified version of the data used the diversity and Random Forest analysis

**File name:** Supplementary Data 3

**Description:** Modified version of the data used the Random Forest analysis
