# Supplementary material for: Gut metagenome profile of the Nunavik Inuit youth is distinct from industrial and non-industrial counterparts
Source: Commun Biol. 2022 Dec 24;5:1415. doi: 10.1038/s42003-022-04372-y (PMC9790006; doi:10.1038/s42003-022-04372-y)
Supplement: Supplementary file 6 — Reporting summary [file 42003_2022_4372_MOESM6_ESM.pdf]

nature portfolio

Corresponding author(s): ROUSSEAU Elsa

Last updated by author(s): 2022/11/17

Reporting Summary

Nature Portfolio wishes to improve the reproducibility of the work that we publish. This form provides structure for consistency and transparency in reporting. For further information on Nature Portfolio policies, see our Editorial Policies and the Editorial Policy Checklist.

Statistics

For all statistical analyses, confirm that the following items are present in the figure legend, table legend, main text, or Methods section.

n/a	Confirmed	
☐	☒	The exact sample size (*n*) for each experimental group/condition, given as a discrete number and unit of measurement
☐	☒	A statement on whether measurements were taken from distinct samples or whether the same sample was measured repeatedly
☐	☒	The statistical test(s) used AND whether they are one- or two-sided<br>*Only common tests should be described solely by name; describe more complex techniques in the Methods section.*
☐	☒	A description of all covariates tested
☒	☐	A description of any assumptions or corrections, such as tests of normality and adjustment for multiple comparisons
☐	☒	A full description of the statistical parameters including central tendency (e.g. means) or other basic estimates (e.g. regression coefficient) AND variation (e.g. standard deviation) or associated estimates of uncertainty (e.g. confidence intervals)
☐	☒	For null hypothesis testing, the test statistic (e.g. *F*, *t*, *r*) with confidence intervals, effect sizes, degrees of freedom and *P* value noted<br>*Give P values as exact values whenever suitable.*
☒	☐	For Bayesian analysis, information on the choice of priors and Markov chain Monte Carlo settings
☐	☒	For hierarchical and complex designs, identification of the appropriate level for tests and full reporting of outcomes
☒	☐	Estimates of effect sizes (e.g. Cohen's *d*, Pearson's *r*), indicating how they were calculated

Our web collection on statistics for biologists contains articles on many of the points above.

Software and code

Policy information about availability of computer code

Data collection	Comparison groups were selected using the curatedMetagenomicData R package (v1.16.1).
Data analysis	Taxonomic and functional profiling were performed using MetaPhlan 3.0 and HUMAnN 3.0 respectively.<br>Diversity analysis were performed using R (v3.6.3) and R packages breakaway (v4.7.3) and vegan (v2.5-7). UMAP representation was performed using umapr R package (v0.0.0.9). Statistical analysis was performed using vegan and ggpubr (v0.4.0). Random Forest classification was performed using the scikit-learn python library (v0.24). Graphical representation were performed using gglopt2 (v3.3.3). Heatmap representation of raysurveyor analysis were perfomed using python (3.7) and python packages scipy (v1.4.1), seaborn (v0.11.1), numpy (v1.19.2), pandas (v1.1.3). Code for statistical and generate graphs are available at https://github.com/corbeillab/GutMetagenomeOfTheNunavikYouth

For manuscripts utilizing custom algorithms or software that are central to the research but not yet described in published literature, software must be made available to editors and reviewers. We strongly encourage code deposition in a community repository (e.g. GitHub). See the Nature Portfolio guidelines for submitting code & software for further information.

Data

Policy information about availability of data

All manuscripts must include a data availability statement. This statement should provide the following information, where applicable:

- Accession codes, unique identifiers, or web links for publicly available datasets
- A description of any restrictions on data availability
- For clinical datasets or third party data, please ensure that the statement adheres to our policy

The data that support the findings of this study were used under copyright agreement and so are not publicly available. In accordance with the First Nations principles of ownership, control, access, and possession (OCAP®), the Nunavik Regional Board of Health and Social Services is the owner of the data and biological samples collected during the Qanuilirpitaa?2017 health survey on behalf of the Inuit population of Nunavik. Any request for data access should be addressed to the Qanuilirpitaa? 2017 Data Management Committee (email: nunavikhealthsurvey@ssss.gouv.qc.ca) that oversees the management of the survey data and biological samples.

Human research participants

Policy information about studies involving human research participants and Sex and Gender in Research.

Reporting on sex and gender	Samples were donated by 186 female and 93 males participants, during the Nunavik Inuit Health Survey2017 (Q2017?) https://www.nrbhss.ca/en/health-surveys-0. The staff of the Q2017 Nunavik Inuit Health Survey collected the informed consents from all participants.<br>Sex can have minor effect on gut microbiome structure, we tested its effect on beta-diversity using PERMANOVA.
Population characteristics	Study participants are all residents of Nunavik. 146 participants originated from Ungava Bay communities, and 133 from Hudson Bay communities. 275 participants declared being of Inuit descent, while, one was Caucasian , and three selected other as ethnicity. Study participants were aged between 16 and 30 year old.
Recruitment	Participants were recruited during the Qanuilirpitaa? 2017 (Q2017) Nunavik Inuit Health Survey. Target participants invited to participate to this study were all Nunavik permanent resident 16 and over. In the 16-30 year old cohort that was selected for this study, more female donated samples. for more information on recruitment see the Q2017 ? health survey methological report,
Ethics oversight	This study was approved by the research ethics committee of the Centre Hospitalier Universitaire de Québec (CHUQ), committee number FWA00000329 and IRB00001242. The study IRB approval # is MP-20-2019-4511. The staff of the Qanuilirpitaa? 2017 (Q2017) Nunavik Inuit Health Survey collected the informed consents from all participants. Moreover, the Data Management Committee of the Qanuilirpitaa? 2017 Nunavik Inuit Health Survey and the authors of this article have built a continuous process whereby mutual interests of the communities and researchers are addressed.

Note that full information on the approval of the study protocol must also be provided in the manuscript.

Field-specific reporting

Please select the one below that is the best fit for your research. If you are not sure, read the appropriate sections before making your selection.

☒ Life sciences ☐ Behavioural & social sciences ☐ Ecological, evolutionary & environmental sciences

For a reference copy of the document with all sections, see nature.com/documents/nr-reporting-summary-flat.pdf

Life sciences study design

All studies must disclose on these points even when the disclosure is negative.

Sample size	In the context of the Qanuilirpitaa? 2017 (Q2017) Nunavik Inuit Health Survey, sample size had to produce reliable estimates for two age groups 16-30 year old and 31 and over. For our metagenome profile study, the 16-30 year old cohort was chosen for analysis. This age group represent 44% of the 12,488 inhabitants of Nunavik. Participants were selected using simple random sampling without replacement. This is decribed here https://www.nrbhss.ca/en/health-surveys-0
Data exclusions	DNA was extracted for 283 feces samples. 4 did not yield sufficient gDNA. 279 samples were successfully sequenced and anlaysed.
Replication	Our study was observational. No replication was performed. . Any request for data access should be addressed to the Qanuilirpitaa? 2017 Data Management Committee (email: nunavikhealthsurvey@ssss.gouv.qc.ca) that oversees the management of the survey data and biological samples.
Randomization	As our study was observational and only data on age, region and sex were used, no randomization was necessary.

Blinding | Our study was not interventional, therefore, blinding was not relevant. To ensure anonymity, a unique identification number has been assigned to each participant to the Q2017? survey.

Reporting for specific materials, systems and methods

We require information from authors about some types of materials, experimental systems and methods used in many studies. Here, indicate whether each material, system or method listed is relevant to your study. If you are not sure if a list item applies to your research, read the appropriate section before selecting a response.

Materials & experimental systems

n/a	Involved in the study
☒	☐ Antibodies
☒	☐ Eukaryotic cell lines
☒	☐ Palaeontology and archaeology
☒	☐ Animals and other organisms
☒	☐ Clinical data
☒	☐ Dual use research of concern

Methods

n/a	Involved in the study
☒	☐ ChIP-seq
☒	☐ Flow cytometry
☒	☐ MRI-based neuroimaging
